# On the Mechanism of Electrocatalytic Carbon–Carbon Coupling of Conjugated Aromatic Aldehydes on Cu Cathodes

**DOI:** 10.1021/acscatal.4c08004

**Published:** 2025-05-26

**Authors:** Hongwen Chen, M. Ussama, Jayendran Iyer, Sungmin Kim, M. Ali Haider, Rachit Khare, Johannes A. Lercher

**Affiliations:** † Department of Chemistry and Catalysis Research Center, 9184 Technical University of Munich, 85748 Garching, Germany; ‡ Renewable Energy and Chemicals Laboratory, Department of Chemical Engineering, 28817 Indian Institute of Technology Delhi, 110016 New Delhi, India; § Institute for Integrated Catalysis, 6865 Pacific Northwest National Laboratory, Richland, Washington 99354, United States; ∥ Indian Institute of Technology Delhi-Abu Dhabi, Khalifa City B, Abu Dhabi MZ29, UAE

**Keywords:** biomass conversion, electrochemical hydrogenation, conjugated aromatic aldehydes, electroreductive carbon−carbon coupling, Cu cathodes

## Abstract

In this work, we show that Cu effectively catalyzes the electroreductive C–C coupling of *only* conjugated aromatic aldehydes (like benzaldehyde). We demonstrate that C–C coupling is not observed for aliphatic aldehydes (like pentanal), nonconjugated aromatic aldehydes (like hydrocinnamaldehyde), or conjugated nonaromatic aldehydes (like crotonaldehyde). The fact that only conjugated aromatic aldehydes undergo C–C coupling on Cu points to the importance of their planar structure and, consequently, their strong interaction with the metal surface. This direct interaction enables electron transfer that eventually leads to the Eley–Rideal (ER)-type reaction with the electrophilic carbon of the reacting physisorbed aldehyde molecule. Two additional factors are indispensable for the C–C coupling pathway: (i) the preferential first hydrogenation of the carbonyl oxygen resulting in the formation of a hydroxy intermediate (i.e., ArCHOH*), and (ii) a relatively slow second H addition resulting in a stable hydroxy intermediate on the surface. In contrast, when other metals or nonconjugated aldehydes are involved, preferential hydrogenation of the carbonyl carbon, fast second H addition, or a high intrinsic barrier for C–C bond formation inhibits the C–C coupling pathway.

## Graphical abstract

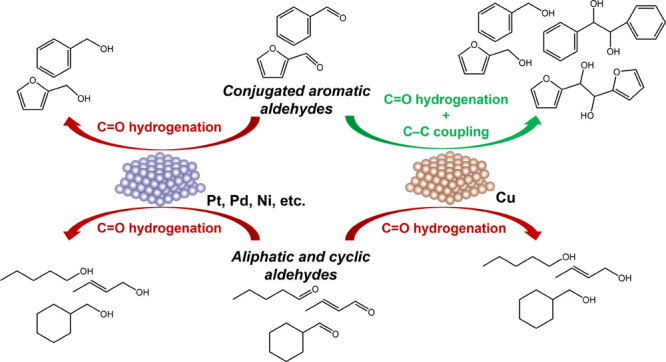

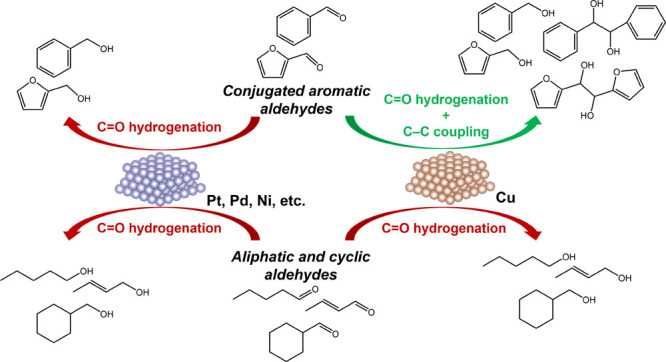

## 1 Introduction

Biomass is an abundant and renewable resource that sequesters the carbon (CO_2_) present in the atmosphere. Therefore, in recent years, significant efforts have been directed toward developing technologies to efficiently convert biomass to fuel-range hydrocarbons, fine chemicals, and other high-value products.^1–5^ However, due to the highly oxygenated and functionalized character of biomass, upgrading requires hydrogenation, carbon–carbon coupling, and subsequent oxygen removal to improve its stability or to increase its energy density.^6–11^

The two key technologies proposed for hydrogenating biomass-derived platform chemicals are thermocatalytic hydrogenation (TCH) and electrocatalytic hydrogenation (ECH). TCH of biomass requires elevated H_2_ pressures and relatively high temperatures.^12,13^ ECH, on the other hand, generates hydrogen in situ from water, eliminating the need for an external H_2_ supply.^14,15^ ECH offers additional advantages over TCH, such as milder operating conditions and integration with the current energy grid.^15,16^ Moreover, if the electricity is generated from a renewable power source, the ECH strategy provides a green, environment-friendly approach to upgrading biomass.

Carbonyl (C=O) functional groups, abundantly present in bio-oils in the form of aldehyde and ketone motifs (often aromatic in nature),^17,18^ are prone to polymerization during the handling and storage of bio-oils. Therefore, their hydrogenation (to the corresponding alcohols) is necessary to stabilize the bio-oils. Several studies have, therefore, focused on the ECH of biomass-derived aldehydes.^16,19–23^ In addition to C=O hydrogenation, carbon–carbon coupling of carbonyl compounds, also referred to as *pinacol coupling*, is an important strategy to upgrade biomass. The C–C coupling products thus obtained find their use in pharmaceutical, transportation and chemical industries.^24,25^ For example, the C–C coupling of furfural or benzaldehyde forms oxygenates with 10–14 carbon atoms, which, upon subsequent ring-opening, oxygen removal, and saturation, can be utilized as biodiesel additives.^24,26^ While pinacol coupling has long been known,^27^ achieving C–C coupling of aldehydes and ketones electrochemically has garnered widespread attention more recently, owing to its greener nature.^28–33^

The ECH of aromatic and aliphatic aldehydes on several metal-based catalysts predominantly results in the C=O hydrogenation.^34–37^ For example, Song et al. demonstrated that ECH of benzaldehyde on carbon-supported Pd, Pt and Rh nanoparticles primarily forms benzyl alcohol as the product.^22^ Yang et al. also investigated the ECH of benzaldehyde on carbon felt supported Pd nanoparticles and observed benzyl alcohol as the primary product.^38^ Cu-based catalysts, on the other hand, have been shown to promote C–C coupling during the ECH of aromatic aldehydes like benzaldehyde and furfural. Chadderdon et al. demonstrated that furfural ECH on Cu follows two distinct pathways, C=O hydrogenation to furfuryl alcohol and C–C coupling to hydrofuroin.^39^ Yu et al. also observed C–C coupling during benzaldehyde ECH on bimetallic Pd–Cu catalysts with a Faradaic efficiency of ∼63%.^24^ We have recently reported on the ability of Cu nanoparticles, supported on carbon, to catalyze both the C=O hydrogenation of benzaldehyde to benzyl alcohol and its C–C coupling to hydrobenzoin.^40,41^

The electroreductive C–C coupling of aldehydes on metal cathodes is typically understood to proceed via a Langmuir–Hinshelwood (LH)-type reaction between two surface-bound ketyl radical intermediates. Anibal et al. proposed that the stability of the ketyl radical could serve as a descriptor for C–C coupling reactions.^42^ However, by combining kinetic and isotopic studies with first-principles density-functional theory (DFT) calculations, we have suggested that, in the case of benzaldehyde ECH on Cu/C, the surface-bound hydroxy intermediate reacts with a solvated physisorbed benzaldehyde molecule, in an ER-type reaction, to form the C–C coupling product (i.e., hydrobenzoin).^40,41^

While both C–C coupling and C=O hydrogenation pathways have been investigated mechanistically, both experimentally and computationally, a clear understanding of the factors that promote the C–C coupling reactions on metal cathodes is still lacking. In this work, we present a detailed investigation of the ECH of various aliphatic and aromatic aldehydes on carbon-supported nanoparticles of several base metals (Cu, Co and Ni) and noble metals (Pt, Pd, Ru, Au and Rh). Our findings show that Cu effectively catalyzes both electrochemical C=O hydrogenation and electroreductive C–C coupling only for conjugated aromatic aldehydes (like benzaldehyde). In contrast, the ECH of aliphatic, nonconjugated aromatic, or conjugated nonaromatic aldehydes leads solely to the C=O hydrogenation product. Combining kinetic studies, aqueous-phase calorimetry, and first-principles molecular simulations, we discuss the mechanistic factors that facilitate C–C bond formation on Cu cathodes.

## 2 Materials and Methods

### 2.1 Chemicals

All chemicals, viz., benzaldehyde (≥99.5% purity), propanal (97% purity), pentanal (≥98% purity), butanal (≥99.5% purity), heptanal (≥97% purity), *trans*-cinnamaldehyde (99% purity), hydrocinnamaldehyde (≥95% purity), 1,2,3,6-tetrahydrobenzaldehyde (cyclohexenal; 97% purity), cyclohexanecarbaldehyde (cyclohexanal; anhydrous, 99.5% purity), furfural (99% purity), 4-methylbenzaldehyde (≥97% purity), 4-methoxybenzaldehyde (≥98% purity), crotonaldehyde (≥99% purity), copper­(II) acetate (99.99% purity), palladium­(II) acetate (97% purity), ruthenium­(III) chloride hydrate (≥99.9% purity), rhodium­(III) nitrate hydrate (∼36% Rh basis), gold­(III) chloride trihydrate (≥99.9% purity), nickel­(II) nitrate (99.999% purity), cobalt­(II) nitrate hexahydrate (≥99.9% purity), acetic acid (≥99.7% purity), sodium acetate (≥99.0% purity), sodium hydroxide (≥98% purity), sodium chloride (≥99% purity), 2-propanol (anhydrous, 99.5% purity), acetone (≥99.9% purity), diphenyl ether (≥99% purity), and ethyl acetate (≥99.5% purity), were purchased from Sigma-Aldrich and used without further purification. Deionized (DI) water (18.2 MΩ·cm) was used to prepare all aqueous solutions.

### 2.2 Catalyst Synthesis

All catalysts were prepared via an incipient wetness impregnation method adapted from previous publications,^21,22,41^ maintaining a similar loading of different metals (∼5 wt %) on a Vulcan XC-72R carbon black (QuinTech) support. The metal precursors were dissolved in either DI water or acetone. After impregnation, the slurries were dried at 383 K overnight. The dried powder was heated to temperatures ranging between 453 and 673 K for 2–4 h under ∼100 mL·min^–1^ N_2_ flow, followed by reduction at temperatures ranging between 473 and 673 K for 2–4 h under ∼100 mL·min^–1^ H_2_ flow. The specific conditions employed for the synthesis of different carbon-supported metal catalysts are summarized in Table S1. Pt/C catalyst (5 wt % loading, dry basis) was purchased from Sigma-Aldrich and was used as acquired without further modification.

### 2.3 Carbon Felt Pretreatment

The carbon felt (Sigma-Aldrich, cut into 3.0 cm × 1.5 cm pieces) was ultrasonically treated sequentially in acetone, DI water, and then acetone again for at least 30 min each at ambient temperature. Finally, the carbon felt was dried in an oven at ∼333 K for 12 h and stored for further use.

### 2.4 Working Electrode Preparation

To prepare the carbon felt working electrode, approximately 10 mg of the as-synthesized catalyst was dispersed in a 1:1 mixture of 2-propanol and DI water. This mixture was subjected to ultrasonication for about 30 min under ambient conditions to create an ink. The ink was then applied to both sides of the pretreated carbon felt. Finally, the carbon felt electrode was placed overnight in an oven at ∼333 K.

### 2.5 Nafion Membrane Pretreatment

A Nafion 117 proton exchange membrane (Ion Power) was immersed in ∼3 vol % H_2_O_2_ solution at 353 K for at least 1 h. Afterward, the membrane was immersed in DI water at 353 K for 2 h. Then, it was placed in a 1 M H_2_SO_4_ solution at 353 K for another 1 h. Finally, the membrane was thoroughly washed several times with DI water and stored in DI water under ambient conditions for subsequent use.

### 2.6 Electrolyte Composition

This work utilized two types of electrolyte solutions for conducting experiments. The acidic electrolyte, with a pH of 4.6, consisted of ∼0.75 M sodium acetate and ∼0.75 M acetic acid, and is referred to as “∼1.5 M acetate buffer solution (pH ≈ 4.6)”. The alkaline electrolyte, with a pH of 11.3, was composed of ∼0.01 M NaOH and ∼0.99 M NaCl, and is referred to as “∼1 M NaOH/NaCl electrolyte solution (pH ≈ 11.3)”.

### 2.7 Electrochemical Measurements

All electrochemical measurements were conducted using a BioLogic VSP-300 workstation. A two-compartment batch electrocatalysis cell was utilized for all electrochemical experiments. The cathode and anode compartments were separated by the Nafion membrane. The design and working of the electrochemical cell has been documented elsewhere.^41^ A 3.0 cm × 1.5 cm carbon felt coated with the carbon-supported metal catalyst was employed as the working electrode. A double-junction leakless miniature Ag/AgCl electrode (eDAQ) served as the reference electrode, while a Pt wire (Sigma-Aldrich) was used as the counter electrode. Prior to the reaction, the cathode was polarized at −40 mA for a minimum of 20 min to ensure the complete reduction of the metal nanoparticles.

The reference electrode was calibrated against a reversible hydrogen electrode (RHE) and all potential values herein are reported relative to the RHE, per the following equation.

1 $$E_{RHE}=E_{Ag/AgCl}+0.197+0.0591\cdot pH$$

where *E*_RHE_ and *E*_Ag/AgCl_ are electrode potentials relative to the RHE and Ag/AgCl electrode, respectively.

All electrochemical experiments were conducted under ambient conditions. A constant flow of approximately 25 mL·min^–1^ of N_2_ was bubbled through the catholyte throughout the reaction to remove dissolved gases. The electrolyte solution was continuously stirred at a speed of 500 rpm. The solution resistance between the working and the reference electrodes was measured using potentiostatic electrochemical impedance spectroscopy (PEIS) and was compensated by the electrochemical workstation to ∼85%.

During the electrochemical experiment, aliquots of approximately 1 mL were periodically withdrawn from the catholyte. The organic compounds in the aqueous catholyte were extracted with 0.5 mL ethyl acetate. The extract was then mixed with an additional 0.5 mL of ethyl acetate containing 20 mM diphenyl ether as an external standard. The extracted organic compounds were analyzed using an offline gas chromatograph coupled with a mass spectrometer (Agilent Technologies 7890B GC/5977A MSD). Refer to the Supporting Information for details regarding the calculations for estimating conversion, Faradaic efficiency (FE), and Faradaic selectivity.

### 2.8 Aqueous-phase Calorimetry Measurements

Aqueous-phase heat of adsorption of organic substrates on carbon-supported metal nanoparticles (under ambient conditions) was estimated using a Setaram C80 calorimeter. For this, approximately 1 mL of the aqueous electrolyte solution containing a specific concentration of the organic substrate was added to the calorimetry cell along with about 20 mg of the catalyst powder. A separate aliquot of approximately 1 mL of the same aqueous electrolyte solution, containing the same concentration of the organic substrate but without the catalyst, was used as the reference.

Equilibrium adsorption amount (*q*_e_) was determined using the following equation

2 $$q_{e}=\frac{\left(C_{0}-C_{e}\right)\cdot V}{m_{cat}}$$

where *C*_0_ and *C*_e_ are the initial and equilibrium concentrations, respectively, *V* is the volume, and *m*_cat_ is the mass of the catalyst.

### 2.9 Computational Details

All quantum chemical simulations were performed on a periodic model using either CP2K electronic structure and molecular dynamics software package (v2022.2),^43^ or the Vienna ab initio simulation package (VASP).^44–47^ Refer to the Supporting Information for details on the computational methods employed.

## 3 Results and Discussion

Carbon-supported metal catalysts (with ∼5 wt % metal loading) were synthesized via an incipient-wetness impregnation method using the corresponding metal salts as the metal source and Vulcan carbon black as the support.^21,22,41^ Refer to the Materials and Methods section for details regarding catalyst synthesis. The synthesized materials were reduced in H_2_ to ensure the formation of metallic nanoparticles, confirmed for example, for Cu/C by X-ray absorption spectroscopy (XAS) measurements.^41^ Additionally, prior to the electrochemical reactions, the cathode was polarized at −40 mA for a minimum of 20 min to ensure the complete reduction of the metal nanoparticles.

### 3.1 ECH of Benzaldehyde on Carbon-Supported Metals

First, let us examine the ECH of benzaldehyde, an aromatic aldehyde, on different carbon-supported base metals (Cu, Ni and Co) and noble metals (Pd, Pt, Ru, Au and Rh) in acidic media (∼1.5 M sodium acetate/acetic acid buffer solution, pH ≈ 4.6). Figure 1a presents the initial product formation rates (measured in terms of H consumption) and the overall Faradaic efficiencies toward organic conversion (FEs) obtained during the ECH of benzaldehyde on different metal cathodes. The FEs toward individual product formation are summarized in Table S2. We see that Pd/C exhibited the highest total H consumption rate (∼14.7 μmol_H_·g_Pd_^–1^·s^–1^) for benzaldehyde ECH among all the metals investigated. However, the FE on Pd was only about 58%. Other noble metals, with the exception of Au, also displayed high total H consumption rates but low FEs (less than 30%) under these reaction conditions. The low FEs on noble metals are attributed to the high overpotential (*E*_ext_ = −0.5 V), which typically leads to enhanced H_2_ formation rates.

**Figure 1:**
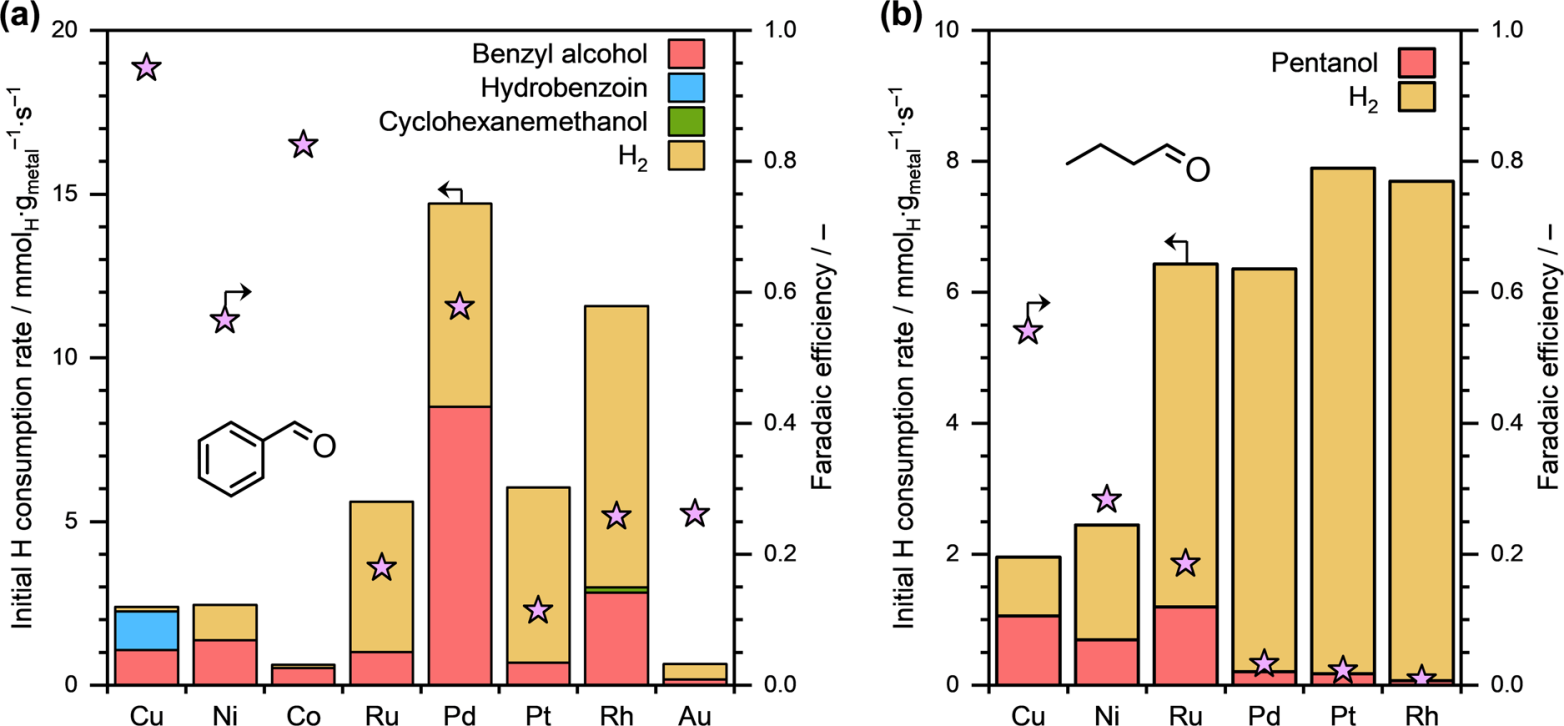
Initial H consumption rates toward different products and Faradaic efficiencies toward organic conversion during the ECH of (a) benzaldehyde and (b) pentanal on various carbon-supported metal cathodes. Reaction conditions: ∼10 mg catalyst, ∼20 mM organic substrate, *E*_ext_ = −0.5 V, ∼1.5 M sodium acetate/acetic acid buffer solution (pH ≈ 4.6), ambient temperature and pressure.

Notably, benzyl alcohol was the only product detected for all noble metals that we investigated, except for Rh, which, in addition to hydrogenating the C=O group, also catalyzed the complete hydrogenation of the aromatic ring resulting in the formation of small amounts of cyclohexanemethanol (see Figure 1a). This means that C–C coupling product (i.e., hydrobenzoin in this case) wasnot observed with these noble metal cathodes under the investigated reaction conditions. It is also important to note that the absence of C–C coupling cannot be attributed to a low FE and, consequently, low surface organic coverage. To confirm this, we conducted the ECH of benzaldehyde on Pd/C and Ru/C at *E*_ext_ = −0.2 V and the results are presented in Figure S1. Even under these reaction conditions that resulted in a FE of ∼100% on Pd/C and ∼89% on Ru/C, benzyl alcohol remained the only product detected.

In contrast to the noble metals, the investigated base metals, viz., Co, Ni and Cu, exhibited relatively lower total H consumption rates during the ECH of benzaldehyde. However, the FEs on these base metals were significantly higher, particularly for Cu, which exhibited almost 94% FE toward benzaldehyde conversion under the investigated reaction conditions (see Figure 1a). These high FEs indicate a relatively high surface coverage of the organic substrate, which results in relatively lower H* coverage and, consequently, lower HER rates. Remarkably, in addition to hydrogenating the C=O group of benzaldehyde to form benzyl alcohol, Cu also catalyzed the C–C coupling of benzaldehyde, leading to the formation of hydrobenzoin with a Faradaic selectivity of approximately 46%. However, in contrast to Cu, Ni and Co catalyzed only the C=O hydrogenation of benzaldehyde to form benzyl alcohol under these reaction conditions. Note that the FE on Co/C (∼83%) was comparable to that on Cu/C (∼94%) suggesting high surface coverage of the organic substrate in both cases. These results, therefore, clearly suggest that the occurrence of C–C coupling for an organic substrate is not directly related to its surface coverage.

### 3.2 ECH of Pentanal on Different Metals

Next, we performed the ECH of pentanal, an aliphatic aldehyde, on various carbon-supported base- and noble-metals under similar reaction conditions (i.e., pH ≈ 4.6 and *E*_ext_ = −0.5 V). Figure 1b shows the initial product formation rates (measured in terms of H consumption) and FEs obtained during the ECH of pentanal on different metal cathodes. The FEs toward individual products are reported in Table S3. Similarly to the benzaldehyde ECH experiments, the noble metals (Pt, Pd, Rh and Ru) exhibited relatively high total H consumption rates but low FEs under these reaction conditions. The base metals (Cu and Ni), on the other hand, showcased relatively lower total H consumption rates but relatively higher FEs during the ECH of pentanal (see Figure 1b). Remarkably, pentanol (the C=O hydrogenation product) was the only product detected, indicating that C–C coupling was not catalyzed on any of the investigated metals during the ECH of pentanal under the applied reaction conditions.

### 3.3 ECH of Other Aliphatic and Aromatic Aldehydes on Cu/C

To further understand the differences in product selectivities obtained during the ECH of benzaldehyde (an aromatic aldehyde) and pentanal (an aliphatic aldehyde), we performed the ECH of several other aliphatic and cyclic/aromatic aldehydes on Cu/C under similar reaction conditions (i.e., pH ≈ 4.6 and *E*_ext_ = −0.5 V). Figure 2a presents the initial H consumption rates toward the formation of different products and the FEs obtained during the ECH of several aliphatic and cyclic aldehydes on Cu/C. The FEs toward the formation of individual products are summarized in Table S4.

**Figure 2:**
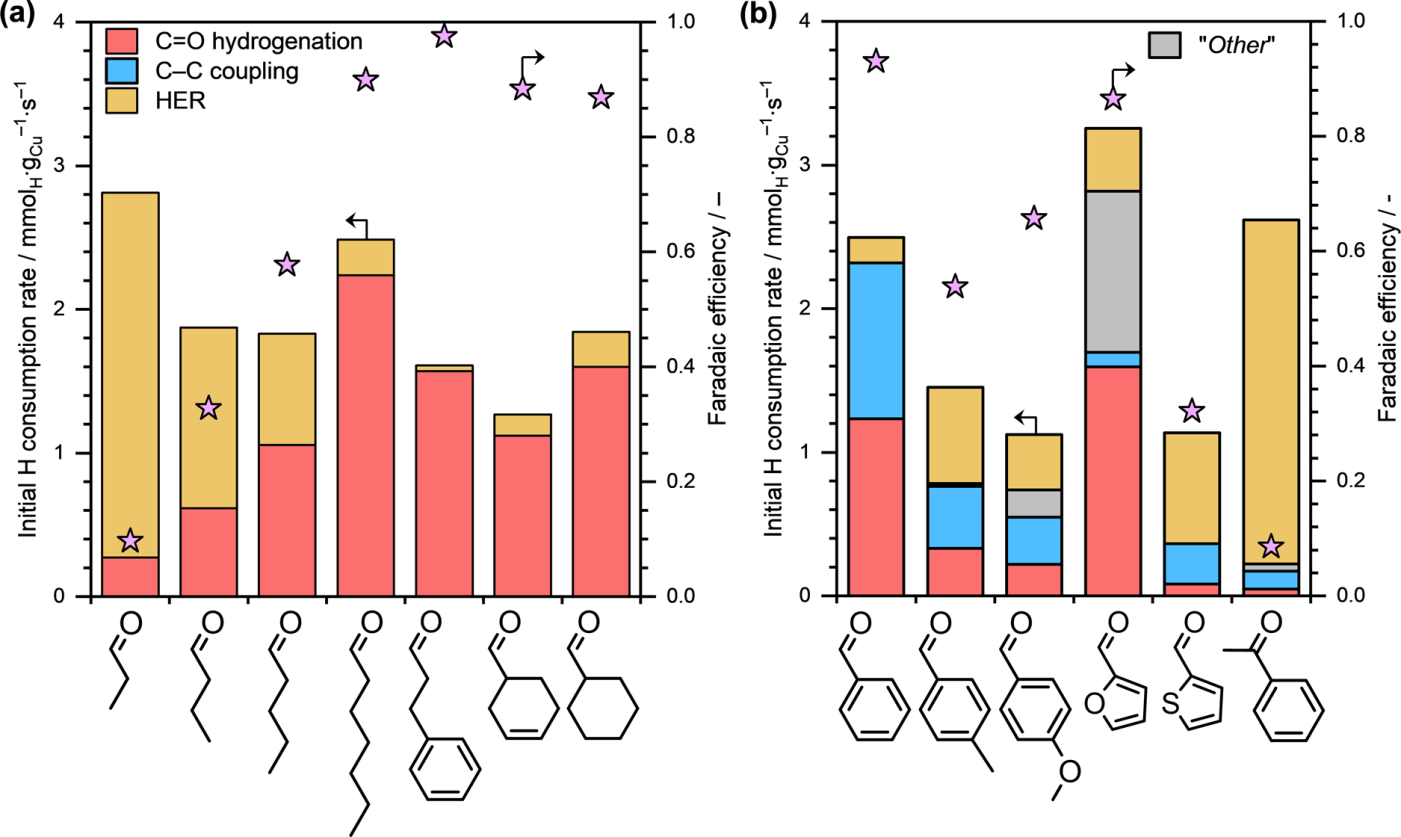
Initial H consumption rates toward different products and Faradaic efficiencies toward organic conversion during the ECH of several (a) aliphatic and cyclic aldehydes and (b) conjugated aromatic aldehydes on Cu/C. Reaction conditions: ∼10 mg catalyst, ∼20 mM organic substrate, *E*_ext_ = −0.5 V, ∼1.5 M sodium acetate/acetic acid buffer solution (pH ≈ 4.6), ambient temperature and pressure. The concentration of “*Other*” products in (b) was estimated from the carbon balance between the reactant converted and products formed. For estimating H consumption toward the formation of “*Other*” products, it was assumed that their formation requires two H^+^/e^–^ per reactant molecule.

First, let us discuss the ECH of four aliphatic aldehydes with varying carbon chain lengths, viz., propanal, butanal, pentanal, and heptanal. While the total H consumption rates were comparable across all cases, the FE increased systematically with the length of the carbon chain—rising from only 10% for propanal ECH to almost 90% during the ECH of heptanal. This systematic increase in the FE with carbon chain length suggests that the surface coverage of the organic substrate increases with the length of the carbon chain. This higher adsorption strength is attributed to stronger interaction between the increasingly long carbon chain and the catalyst surface.^48^ However, irrespective of the interaction strength or the surface coverage, C–C coupling products were not detected in these experiments. Therefore, we conclude that Cu does not catalyze the C–C coupling during the ECH of aliphatic aldehydes.

Figure 2a also shows the product formation rates on Cu/C during the ECH of three cyclic aldehydes, viz*.*, hydrocinnamaldehyde, cyclohexenal and cyclohexanal. The total H consumption rates were similar for these cyclic aldehydes, and the FEs were relatively high. However, even for these cyclic aldehydes, C–C coupling products were not observed. The absence of C–C coupling during the ECH of cyclohexenal and cyclohexanal suggests that the presence of an aromatic ring in the aldehyde, like in the case of benzaldehyde, facilitates the C–C bond formation. The lack of C–C coupling products during the ECH of hydrocinnamaldehyde suggests that the aromatic ring in the aldehyde must be conjugated with the C=O group for C–C coupling to take place.

To confirm this hypothesis, we performed the ECH of several aromatic aldehydes featuring a conjugated aromatic ring and a C=O functional group (referred to as conjugated aromatic aldehydes). Figure 2b presents the product formation rates (in terms of H consumption) and the FEs obtained during the ECH of several conjugated aromatic aldehydes on Cu/C. The product formation rates and FEs obtained during benzaldehyde ECH are also shown for comparison. The corresponding FEs of the formation of individual products are reported in Table S5. First of all, we see that the FEs varied significantly across these experiments, ranging between ∼32% during 3-thiophenecarboxaldehyde ECH to almost 94% for benzaldehyde ECH. This indirectly suggests the surface coverage of the organic intermediates is likely to be substantially different across the studied organic substrates. However, irrespective of the obtained FE or the surface coverage, we observed the formation of both C=O hydrogenation and C–C coupling products during the ECH of these conjugated aromatic aldehydes, although with varying selectivities.

We must note here that in the case of 4-methoxybenzaldehyde and furfural ECH on Cu, the carbon balance between the reactants converted and products formed during their ECH reaction was not complete. This difference is denoted as “*Other*” products in Figure 2b. These byproducts are likely to be either (i) high molecular weight compounds that are adsorbed on the carbon felt electrode or (ii) light hydrocarbons that escaped to the gas phase due to constant bubbling of N_2_ through the electrolyte solution—for example, methylfuran, which has a boiling point of only about 336 K, has been observed as a byproduct during the ECH of furfural on Cu electrodes.^39^

We also performed the ECH of cinnamaldehyde on Cu/C at *E*_ext_ = −0.5 V. Interestingly, during this experiment, although the concentration of cinnamaldehyde in the electrolyte decreased over time, we detected only small amounts (less than 5% of the total carbon converted) of hydrocinnamaldehyde (the C=C hydrogenation product) and cinnamyl alcohol (the C=O hydrogenation product) in the electrolyte. To investigate further, we extracted the organic compounds that may have been adsorbed on the carbon felt electrode and analyzed them with gas chromatography. The resultingchromatogram is presented in the Figure S2. The presence of high molecular weight compounds (evident at long retention times) suggests that C–C coupling products were likely formed during the ECH of cinnamaldehyde on Cu/C.

Lastly, we also performed the ECH of acetophenone, a ketone featuring conjugated aromatic ring and a C=O functional group, on Cu/C and the results are reported in Figure 2b. Notably, the FE during acetophenone ECH on Cu/C was very low (less than 10%). However, we still observed the formation of the C–C coupling product of acetophenone under the applied reaction conditions. Overall, based on these experiments, we conclude that aldehydes and ketones that feature a conjugation between the aromatic ring and the C=O functional group undergo C–C coupling reaction on Cu cathodes.

### 3.4 ECH of Crotonaldehyde on Cu

Crotonaldehyde is an aliphatic aldehyde in which the C=O group is conjugated with a C=C double bond instead of an aromatic ring. Therefore, to understand how the presence of an aromatic ring impacts the C–C coupling ability of Cu, we conducted the ECH of crotonaldehyde on Cu/C. Figure 3 presents the concentration profiles of reactants and products obtained during crotonaldehyde ECH on Cu/C at *E*_ext_ = −0.5 V. The FE for this reaction was ∼76%. We can clearly see that crotonaldehyde ECH on Cu/C resulted in two primary products: crotyl alcohol (the C=O hydrogenation products) and butanal (the C=C hydrogenation product). Notably, the formation of butanal during crotonaldehyde ECH and that of hydrocinnamaldehyde during cinnamaldehyde ECH suggests that Cu also facilitates C=C bond hydrogenation during the ECH reaction.

**Figure 3:**
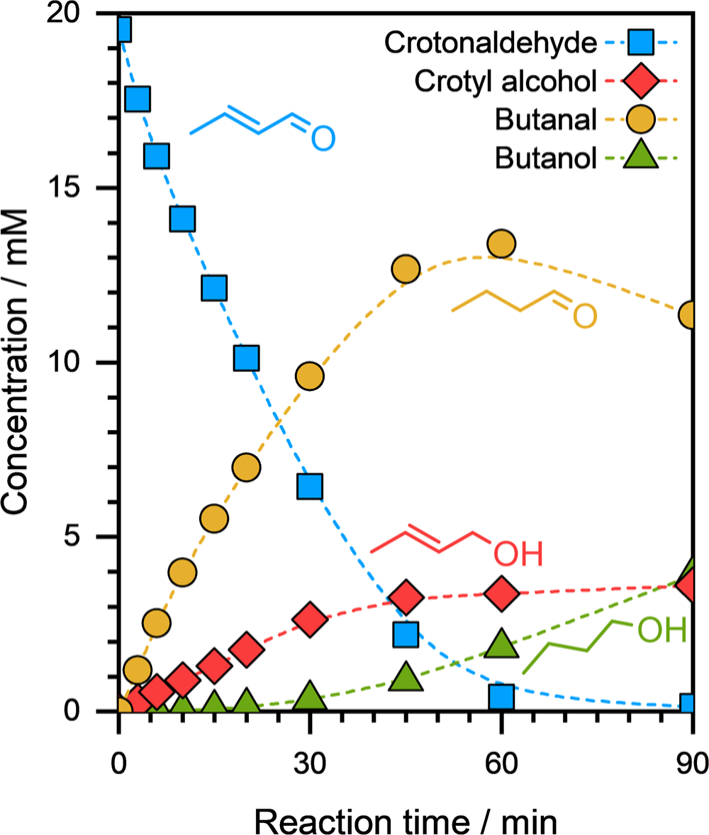
Concentration profiles of reactants and products as a function of reaction time during the ECH of crotonaldehyde on Cu/C. Reaction conditions: ∼10 mg catalyst, ∼20 mM organic substrate, *E*_ext_ = −0.5 V, ∼1.5 M sodium acetate/acetic acid buffer solution (pH ≈ 4.6), ambient temperature and pressure. The dashed lines are visual guides.

As the reaction progressed, butanol was detected as the secondary product of crotonaldehyde ECH on Cu/C (see Figure 3). Moreover, butanal concentration decreased as butanol was being formed suggesting that butanol is likely formed via C=O hydrogenation of butanal. On the other hand, crotyl alcohol concentration remained almost invariant during this period suggesting that hydrogenation of the C=C bond in crotyl alcohol is not catalyzed on Cu under these reaction conditions. Therefore, based on these concentration profiles, we postulate that the C=C bonds that are conjugated with the C=O functional group are more likely to be hydrogenated on Cu. Lastly, no C–C coupling products were observed during crotonaldehyde ECH on Cu/C, clearly suggesting that the presence of an aromatic ring in conjugation with the C=O group is a prerequisite for C–C coupling to occur during ECH of aldehydes on Cu cathodes.

### 3.5 ECH of Aldehydes in Alkaline Media

Alkaline conditions have been suggested to provide a good platform for C–C coupling reactions with high FEs.^26,40,49^ Therefore, we explored ECH of aldehydes on different carbon-supported metals in alkaline conditions (at pH ≈ 11.3). Figure 4a presents the initial product formation rates (measured in terms of H consumption) and FEs during the ECH of benzaldehyde on different base and noble metals in alkaline media. The FEs toward the formation of individual products are summarized in Table S6.

**Figure 4:**
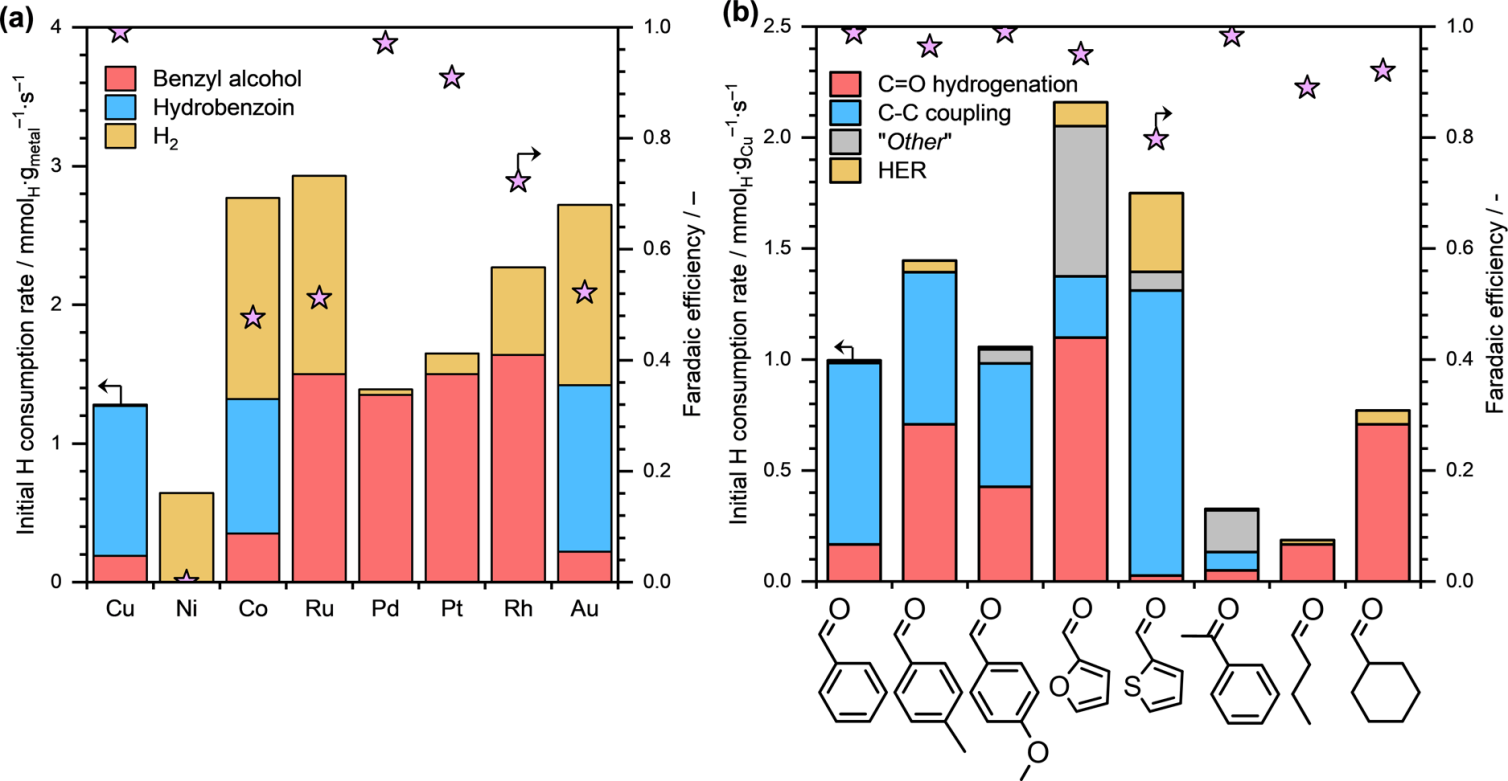
Initial H consumption rates toward different products and the Faradaic efficiencies toward organic conversion during (a) the ECH of benzaldehyde on various carbon-supported metals and (b) various aliphatic and aromatic aldehydes on Cu/C. Reaction conditions: ∼10 mg catalyst, ∼20 mM organic substrate, *E*_ext_ = −0.7 V for Co/C, Au/C and −0.5 V for other metals, ∼1 M NaOH/NaCl electrolyte solution (pH ≈ 11.3), ambient temperature and pressure. The concentration of “*Other*” products in (b) was estimated from the carbon balance between the reactant converted and products formed. For estimating H consumption toward the formation of “*Other*” products, it was assumed that their formation requires two H^+^/e^–^ per reactant molecule.

The total H consumption rates obtained in alkaline media were generally lower than those in acidic media (see Figure 1a). In fact, the net ECH activity on Co/C and Au/C at *E*_ext_ = −0.5 V was negligible. Therefore, we conducted benzaldehyde ECH on Co/C and Au/C at a higher overpotential and the results presented in Figure 4a for Co/C and Au/C were obtained at *E*_ext_ = −0.7 V. The relatively lower ECH activity in alkaline media is attributed to slow H addition kinetics, likely caused by a weaker protonation ability of H_2_O molecules in alkaline media compared to H_3_O^+^ ions in acidic media. The FEs in alkaline conditions were also generally high on all metals with the exception of Ni/C, which exhibited almost no benzaldehyde conversion under the applied reaction conditions. These relatively high FEs are attributed to the low HER rates, caused by the slower H addition kinetics, in alkaline conditions.

While Pd, Rh, Ru and Pt catalyzed benzaldehyde hydrogenation to primarily benzyl alcohol in alkaline conditions, Cu, Co, and Au additionally catalyzed the C–C coupling of benzaldehyde to hydrobenzoin (see Figure 4a). Liu et al. also observed C–C coupling during benzaldehyde ECH on Au, Cu and Co cathodes in ∼1 M KOH electrolyte.^29^ We also noticed that the FE toward organic conversion was significantly higher on Cu/C (almost 99%) than the FE on Co/C or Au/C (only ∼48% and ∼52%, respectively). This is likely because benzaldehyde ECH on Co/C and Au/C was performed at a significantly higher overpotential (i.e., *E*_ext_ = −0.7 V) compared to Cu/C (*E*_ext_ = −0.5 V).

To explore the different selectivities further, we performed the ECH of several conjugated aromatic aldehydes on Cu/C in alkaline conditions and the obtained product formation rates (measured in terms of H consumption) and FEs are presented in Figure 4b. The FEs regarding the formation of individual products are reported in Table S7. The products classified as “*Other*” products in Figure 4b are either high molecular weight compounds that remained adsorbed on the carbon felt electrode or light hydrocarbons that escaped to the gas phase. First of all, as expected, the overall FEs were significantly higher in alkaline conditions as compared to the FEs obtained in acidic conditions. Moreover, similarly to the results in acidic media (see Figure 2b), we observed the formation of both C=O hydrogenation and C–C coupling products during the ECH of conjugated aromatic aldehydes. Notably, the ECH of acetophenone on Cu/C in alkaline media also resulted in significant FE toward the C–C coupling reaction with high overall FE (see Figure 4b). Overall, these results indicate that alkaline conditions promote the C–C coupling pathways during the ECH of conjugated aromatic aldehydes and ketones on Cu/C.

Lastly, we performed the ECH of pentanal, an aliphatic aldehyde, and cyclohexanal, a cyclic aldehyde, on Cu/C in alkaline conditions and results are presented in Figure 4b. The corresponding FEs for the formation of individual products are reported in Table S7. We did not observe C–C coupling products during the ECH of these nonconjugated aldehydes, even in alkaline conditions that have been shown to promote the C–C coupling reaction. Therefore, these series of experiments involving several different classes of aldehydes in both acidic and alkaline media strongly suggest that only conjugated aromatic aldehydes undergo C–C coupling during their ECH on Cu/C.

### 3.6 Mechanism of ECH of Aldehydes on Metal Surfaces

Previously, we have described the mechanism of electrochemical C=O hydrogenation and C–C coupling during benzaldehyde ECH on Cu/C in both acidic and alkaline media.^40,41^ Here, we extend that mechanism and postulate a general mechanism for the C=O hydrogenation and C–C coupling. The proposed mechanism is depicted in Scheme 1. In the proposed mechanism, first, an aldehyde molecule (RCHO) adsorbs reversibly on a vacant site on the metal surface (*). The adsorbed aldehyde (RCHO*) then undergoes first H addition via two distinct pathways: (i) the hydroxy pathway (blue arrows; Scheme 1) in which the carbonyl O is hydrogenated to form a surface hydroxy intermediate (RCHOH*), or (ii) the alkoxy pathway (red arrows; Scheme 1) where the carbonyl C is hydrogenated to form a surface alkoxy intermediate (RCH_2_O*). For C=O hydrogenation pathway, the hydroxy and alkoxy intermediates undergo a second H addition, either at the radical α-C of the hydroxy intermediate or at the radical O of the alkoxy intermediate, to form RCH_2_OH*, which subsequently desorbs to form the alcohol product.

**Scheme 1:**
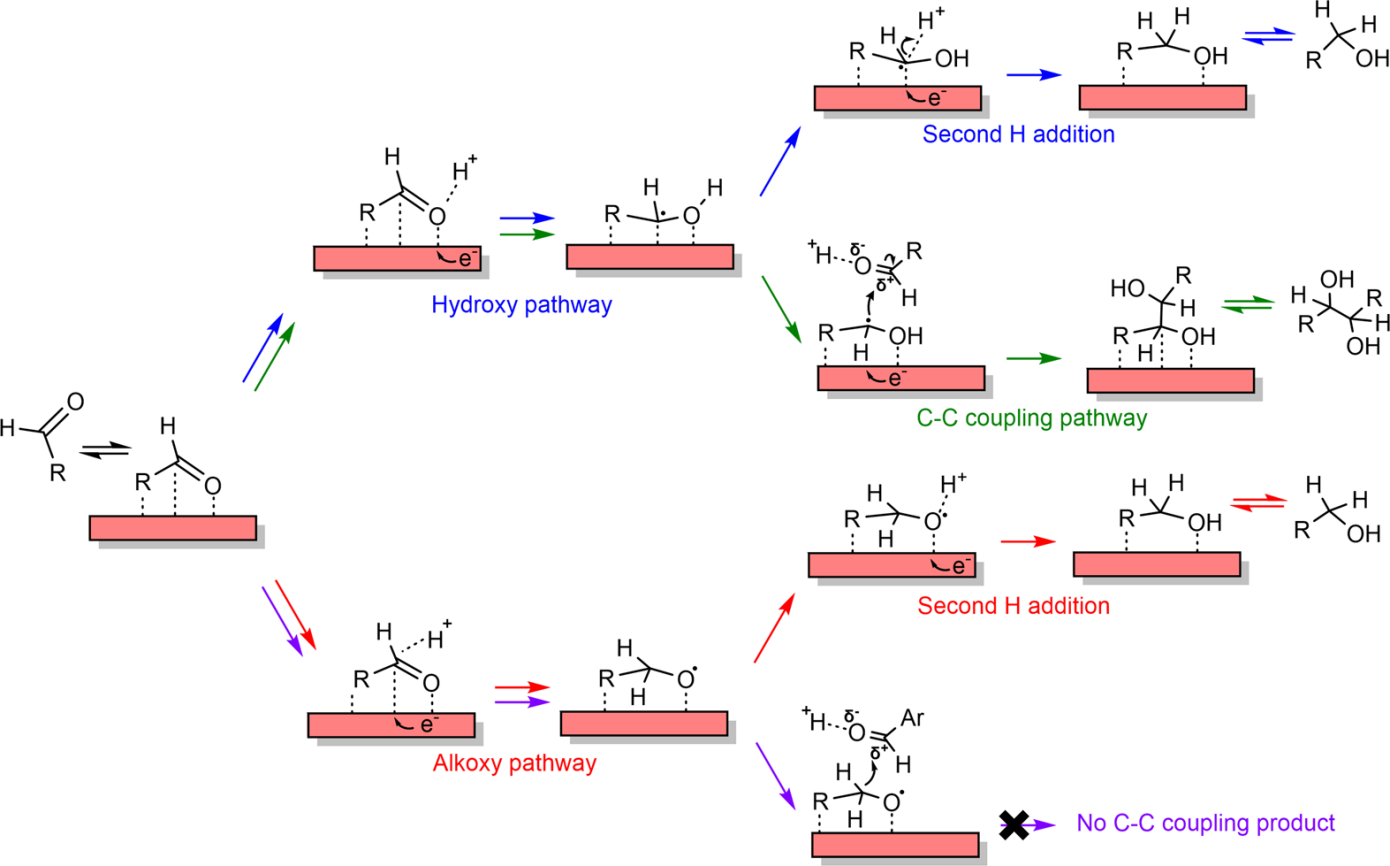
Mechanism of C=O Hydrogenation and C–C Coupling Reactions During the ECH of Aldehydes on a Metal Surface

The C–C coupling during the ECH of aldehydes and ketones has been proposed to proceed via two distinct pathways: (i) an LH-type mechanism that involves the surface recombination of two ketyl radicals, or (ii) an ER-type mechanism (illustrated in Scheme 1; green arrows) wherein the radical α-C of the hydroxy intermediate reacts with the electrophilic carbonyl C of a nearby physisorbed aldehyde molecule (RC^δ+^HO^δ−^). The C–C bond formation is followed by a fast second H addition to form the C–C coupling product. Combining kinetic and isotopic studies with first-principles molecular simulations, we demonstrated that C–C coupling during benzaldehyde ECH on Cu/C occurs via the ER-type mechanism in both acidic and alkaline conditions.^40,41^ Now, we delineate the necessary conditions facilitating C–C coupling based on the postulated mechanism.

The C–C coupling between two aldehyde molecules requires bond formation between the carbonyl C atoms of the two aldehyde molecules, in addition to the hydrogenation of the carbonyl O atoms of each aldehyde molecule. This is true for both LH-type C–C coupling between two ketyl radicals and ER-type C–C coupling mechanism proposed in Scheme 1. The formation of C–C bond, therefore, necessitates that the first H addition must proceed via the hydroxy pathway, i.e., the carbonyl O must be preferentially hydrogenated. In contrast, preferential first H addition to the carbonyl C via the alkoxy pathway will inadvertently saturate the C atom and, consequently, hinder C–C bond formation (purple arrows; Scheme 1). Utilizing isotope labeling studies we demonstrated that for benzaldehyde ECH on Cu/C in acidic media, the first H addition indeed proceeds via the hydroxy pathway.^41^

Next, based on the proposed mechanism, the surface hydroxy intermediate has two options: (i) it can either undergo second H addition to form the C=O hydrogenation product (blue arrows; Scheme 1), or (ii) it can react with another hydroxy intermediate (in the LH-type mechanism) or with a physisorbed benzaldehyde molecule (in ER-type mechanism) to form the C–C coupling product (green arrows; Scheme 1). We also note that a slow second H addition (with respect to the first H addition) will result in a relatively high surface coverage of the hydroxy intermediate. On the other hand, a faster second H addition step would consume the hydroxy intermediate, thus lowering its concentration on the surface. If the C–C coupling is the rate-determining step, then the rate of formation of the C–C coupling product (*r*_C–C_) can be expressed as

3 $$r_{C-C,LH}=k_{C-C,LH}\cdot {\theta_{RCHOH*}}^{2}$$

$$r_{C-C,ER}=k_{C-C,ER}\cdot \theta_{RCHOH*}\cdot a_{RCHO}$$ 4

where *k*_C–C,LH_ and *k*_C–C,ER_ are the kinetic rate constants for the LH-type and ER-type steps, respectively, θ_RCHOH*_ is the surface coverage of the hydroxy intermediate, and *a*_RCHO_ is the activity of aldehyde in the Helmholtz layer. In either case, a low surface coverage of RCHOH* will inhibit the C–C coupling reaction. Therefore, we postulate that a faster second H addition compared to the first H addition will inhibit C–C coupling by consuming the hydroxy intermediate. Based on kinetic measurements and first-principles molecular simulations, we have previously demonstrated that the second H addition is indeed slower than the first H addition during the ECH of benzaldehyde on Cu/C, both in acidic media and in alkaline media.^40,41^

Lastly, we note that even if the first two mechanistic conditions are met, i.e., the first H addition proceeds via the hydroxy pathway and the second H addition is slower as compared to the first H addition, an intrinsically higher activation barrier for the C–C coupling step (compared to that for the second H addition) will still kinetically inhibit the formation of any C–C coupling product. In our previous work, utilizing DFT calculations, we demonstrated that for benzaldehyde ECH on a Cu(111) surface, the activation barrier for the ER-type C–C coupling step was significantly lower than the activation barrier for the second H addition in both acidic and alkaline media.^40,41^

To summarize, we hypothesize that the following mechanistic conditions must be met for the C–C coupling to occur on metal electrodes. *First*, the hydroxy pathway must be the preferred pathway; preferential first H addition to the carbonyl carbon via the alkoxy pathway will saturate the C atom and inhibit C–C coupling. *Second*, the second H addition step must be the rate-determining step for the C=O hydrogenation pathway; a relatively fast second H addition will consume the hydroxy intermediate and primarily form the C=O hydrogenation product. *Third*, the intrinsic activation barrier for C–C coupling step must be lower than that for the second H addition; an intrinsically high barrier for the C–C bond formation transition state will also inhibit the C–C coupling pathway. As mentioned above, in our previous studies,^40,41^ we have shown that all three conditions are met in the case of benzaldehyde ECH on Cu/C resulting in high selectivity toward the C–C coupling product. In the following section, utilizing first-principles molecular simulations, we demonstrate different scenarios that inhibit the C–C coupling reaction.

### 3.7 First-Principles Molecular Simulations

First, let us examine the relative stability of the hydroxy and alkoxy intermediates of benzaldehyde ECH on Cu and Ru. Periodic density-functional theory (DFT) simulations were performed on a system comprising an organic substrate adsorbed on a Cu(111) or a Ru(0001) surface. The Cu(111) facet was employed as it was the most abundant facet observed in the X-ray diffraction patterns of Cu/C.^41^ The Ru(0001) facet was chosen due to its structural similarity to the Cu(111) facet, making it suitable for comparison. The system was solvated with 48 explicit water molecules and a proton (H^+^). A corresponding electron was added to the system to simulate electrochemical reaction conditions. For more details on the employed computational methods, refer to the Materials and Methods section.

The DFT-optimized geometries of an adsorbed benzaldehyde molecule on Cu(111) and Ru(0001) surfaces are presented in Figure S3. Figure 5a presents the top views of the DFT-optimized geometries of the hydroxy (ArCHOH*) and the alkoxy (ArCH_2_O*) intermediates of benzaldehyde ECH on the Cu(111) surface. The corresponding side views are presented in Figure S4. To simulate these intermediates, we added an H atom to either the carbonyl C or the carbonyl O of the adsorbed benzaldehyde molecule (ArCHO*) and relaxed the system to obtain optimized geometries. We calculated the difference in the electronic energies of the hydroxy and the alkoxy intermediates, which is denoted as Δ*E*_OH–CH_. In the case of benzaldehyde ECH on Cu(111) surface, Δ*E*_OH–CH_ was estimated to be approximately −25 kJ·mol^–1^, which means that the hydroxy intermediate is thermodynamically more stable than the alkoxy intermediate. This agrees well with the experimental evidence, which suggests that benzaldehyde ECH on Cu/C proceeds primarily via the hydroxy pathway.^41^

**Figure 5:**
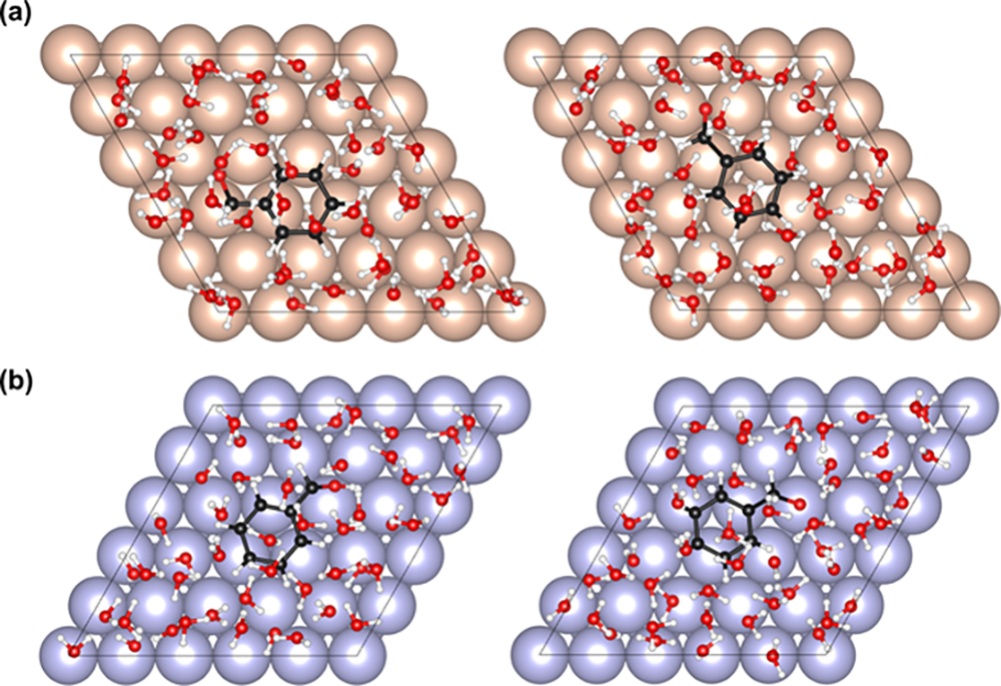
DFT-optimized geometries (top view) of the adsorbed hydroxy intermediates (left panels) and alkoxy intermediates (right panel) on (a) Cu(111) surface and (b) Ru(0001) surface. Cu, orange; Ru, purple; C, black; O, red; H, white. The electrode–electrolyte system comprised 100 metal atoms, organic substrate, 48 explicit water molecules, a proton (H^+^), and a corresponding electron.

Next, we estimated the relative stability of the hydroxy and alkoxy intermediates of benzaldehyde on Ru(0001) surface and the top views of the DFT-optimized configurations are presented in Figure 5b. The corresponding side views are presented in Figure S5. Based on the electronic energies of the optimized configurations, we calculated the Δ*E*_OH‑CH_ to be +46 kJ·mol^–1^ on Ru(0001). Therefore, for benzaldehyde ECH on the Ru(0001) surface, the alkoxy intermediate was found to be thermodynamically more stable than the hydroxy intermediate. Interestingly, Sinha and Neurock also showed through their theoretical simulations that the alkoxy intermediates are more stable during the hydrogenation of C_1_–C_4_ ketones on Ru.^50^ These molecular simulations, therefore, clearly suggest that H addition in the case of benzaldehyde ECH on Ru is likely to proceed via the alkoxy pathway. Consequently, the preferential first H addition to the carbonyl C will inhibit the C–C coupling pathway during benzaldehyde ECH on Ru/C, resulting in benzyl alcohol as the predominant product, as observed experimentally (see Figure 1a).

We also simulated the relative stability of the hydroxy and alkoxy intermediates of crotonaldehyde ECH on Cu(111) surface and the corresponding DFT-optimized configurations are presented in Figure S6. In the case of crotonaldehyde ECH on Cu, the Δ*E*_OH‑CH_ was estimated to be approximately +75 kJ·mol^–1^. In other words, the alkoxy intermediate of crotonaldehyde ECH is thermodynamically more stable than its hydroxy intermediate. Similarly to benzaldehyde ECH on Ru(0001) surface, these computational results suggest that H addition in the case of crotonaldehyde ECH on Cu proceeds via the alkoxy pathway, thus inhibiting C–C coupling pathway. Again, this agrees well with the experimental results, where we observed no C–C coupling during crotonaldehyde ECH on Cu/C (see Figure 3).

The estimated Bader charges on the carbonyl carbon and oxygen atoms of adsorbed benzaldehyde and crotonaldehyde intermediates on Cu(111) and Ru(0001) surfaces are summarized in Table S8. The Bader charge analysis shows that the carbonyl O of benzaldehyde adsorbed on Cu(111) surface exhibits a significantly higher negative charge compared to that on the carbonyl O of benzaldehyde adsorbed on the Ru(0001) surface or the carbonyl O of crotonaldehyde adsorbed on the Cu(111) surface. Concurrently, the carbonyl C of benzaldehyde adsorbed on Cu(111) had a higher positive charge than that of benzaldehyde adsorbed on Ru(0001) or crotonaldehyde adsorbed on Cu(111). The more nucleophilic nature of carbonyl O of benzaldehyde adsorbed on Cu implies stronger electrostatic interaction with protons from the solvent, which would facilitate the hydroxy pathway over the alkoxy pathway.

Next, we simulated the relative stability of the hydroxy and alkoxy intermediates of two nonconjugated aldehydes, viz., cyclohexanal and pentanal, on the Cu(111) surface. The adsorption configurations of benzaldehyde, cyclohexanal and pentanal adsorbed on the Cu(111) surface are compared in Figure S7. We can see that the planar benzaldehyde molecule adsorbs flat at a distance of ∼2.1 Å from the surface of Cu. The nonplanar cyclohexanal and pentanal molecules, on the other hand, adsorb at a significantly longer distance (2.7–2.8 Å) from the surface of Cu. Therefore, these simulations indicate that the interaction of nonconjugated aldehydes like cyclohexanal and pentanal with the surface of Cu is likely weaker than that of benzaldehyde, which interacts strongly via its aromatic ring.

Next, to model the hydroxy and alkoxy intermediates of cyclohexanal and pentanal, we added an H atom to either the carbonyl C or the carbonyl O atom of an adsorbed molecule (RCHO*). However, upon electronic structure relaxation, neither intermediate could be stabilized on the Cu(111) surface. Instead, both intermediates underwent a spontaneous second H addition, directly forming cyclohexanol and pentanol, respectively. These findings, therefore, suggest that the second H addition in the case of cyclohexanal or pentanal ECH on Cu must be fast. This facile second H addition consequently consumes the hydroxy or the alkoxy intermediates and hence suppresses C–C coupling. This result also agrees well with the experimental results, which demonstrated that C–C coupling does not occur during cyclohexanal or pentanal ECH on Cu/C (see Figure 2a).

Finally, we investigated the C–C coupling reaction between a surface hydroxy intermediate (ArCHOH*) and a physisorbed benzaldehyde molecule (ArCHO) on the Ru(0001) surface. For simulating the ER-type C–C coupling reaction, we placed a physisorbed benzaldehyde molecule next to an adsorbed hydroxy intermediate. The C–C bond formation involved a reaction between the electrophilic carbonyl C of the physisorbed benzaldehyde molecule and the radical α-C of the hydroxy intermediate. The optimized geometries (top view) of the initial (IS), transition (TS^‡^) and final state (FS) are presented in Figure 6. The corresponding side views are presented in Figure S8. The electronic energy barrier for the C–C bond formation step was estimated to be approximately 120 kJ·mol^–1^. For comparison, the C–C coupling barrier for a similar C–C coupling step on Cu(111) surface was only 36 kJ·mol^–1^ (both in acidic and alkaline conditions).^41^ These results, therefore, indicate the C–C bond formation on Ru has an intrinsically high energy barrier, and therefore, even if the hydroxy intermediate was stable on the Ru(0001) surface, C–C coupling is unlikely to occur, at least via the ER-type reaction pathway.

**Figure 6:**
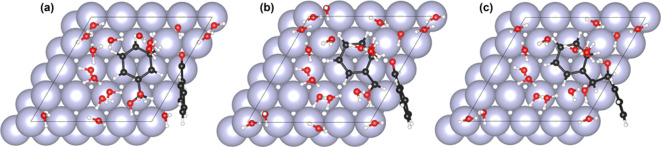
(a) Initial state, (b) transition state, and (c) final state of the C–C bond formation step between a surface hydroxy intermediate and a physisorbed benzaldehyde molecule to form HB*. The simulations were performed on a Ru(1000) surface. Ru, purple; C, black; O, red; H, white. The electrode–electrolyte system comprised 64 metal atoms, organic substrate, 16 explicit water molecules, a proton (H^+^), and a corresponding electron.

We estimated the Bader charges on the hydroxy intermediate of benzaldehyde on both Cu(111) and Ru(0001) surfaces, and the results are summarized in Table S9. The Bader charge analysis shows that the overall charge on the hydroxy intermediate adsorbed on Cu(111) to be lower compared to that on the Ru(0001) surface. We postulate that this enhanced charge transfer from the metal surface to the hydroxy intermediate facilitates ER-type coupling of the hydroxy intermediate with the electrophilic carbon of the reacting physisorbed aldehyde molecule.

Overall, by combining experiments and computational simulations, we postulate that conjugated aromatic aldehydes uniquely undergo C–C coupling on Cu likely because all three mechanistic conditions are met: (i) first H addition to the carbonyl O atom via the hydroxy pathway, (ii) slow second H addition step, and (iii) a low activation barrier for the C–C bond formation.

### 3.8 Aqueous-phase Heat of Adsorption of Aldehydes on Metals

To further understand the unique behavior of conjugated aromatic aldehydes, we examined the adsorption enthalpies of various aldehydes investigated in this work on different metal surfaces. The experimentally determined aqueous-phase adsorption enthalpies (Δ*H*_ads_) of several aliphatic and aromatic aldehydes are compared in Figure 7. Interestingly, the −Δ*H*_ads_ of conjugated aromatic aldehydes, like benzaldehyde, furfural and cinnamaldehyde on Cu/C, was very high, exceeding 145 kJ·mol^–1^. Notably, the adsorption enthalpies of furfural and benzaldehyde on Cu/C were relatively high in alkaline conditions as well. We postulate that as these conjugated aromatic aldehydes are planar, they adsorb flat on the surface of Cu with strong interaction between the surface and the unsaturated, electron-rich aromatic ring, resulting in these high −Δ*H*_ads_ values.

**Figure 7:**
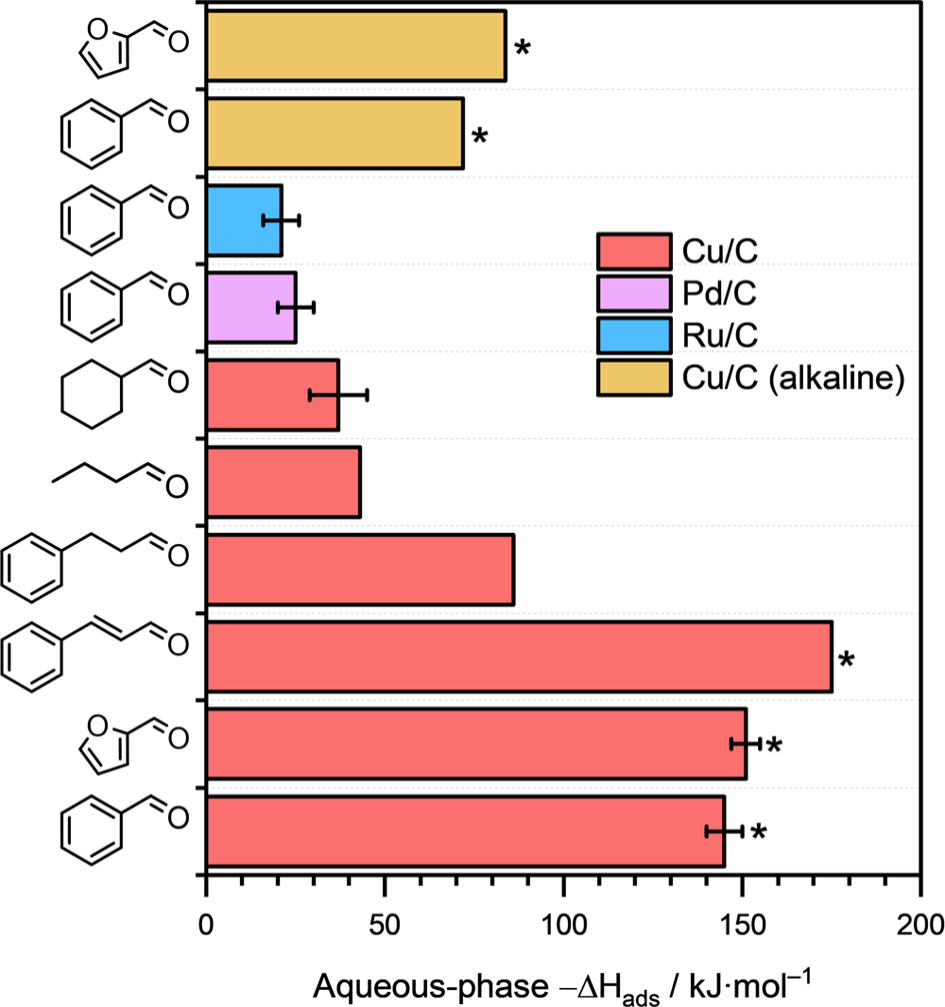
Aqueous-phase heats of adsorption of several aliphatic and aromatic aldehydes on Cu/C (red), Pd/C (purple) or Ru/C (blue) in acidic media and on Cu/C in alkaline media (yellow) under ambient conditions, determined from calorimetry. *C–C coupling occurred during the ECH of these organic substrates under those reaction conditions.

On the other hand, saturated cyclic aldehydes and aliphatic aldehydes are not planar and interact with the Cu surface primarily via their C=O functional groups. As a result, the −Δ*H*_ads_ of such aliphatic aldehydes (like pentanal) and cyclic aldehydes (such as cyclohexanal) on Cu/C were relatively low, less than 50 kJ·mol^–1^. We also determined the adsorption enthalpy of benzaldehyde on Pd/C and Ru/C and the results are also reported in Figure 7. Interestingly, the −Δ*H*_ads_ of benzaldehyde on these metal that did not catalyze C–C coupling reaction was also relatively low, indicating relatively weak bonding of benzaldehyde with the surface of Ru or Pd under these reaction conditions.

These trends in adsorption enthalpies suggest that a strong interaction of the aromatic ring with the surface of Cu likely promotes C–C coupling. However, we must also note here that nonconjugated aromatic aldehydes, like hydrocinnamaldehyde, also exhibited relatively high −Δ*H*_ads_ values on Cu/C (∼86 kJ·mol^–1^), but did not result in any C–C coupling product during their reaction on Cu. Therefore, we further postulate that the conjugation between and aromatic ring and the C=O function group is also necessary for C–C coupling to take place. We speculate that the strong interaction of the organic substrate with the metal’s surface and the conjugation between the aromatic ring and the C=O functional group stabilizes the hydroxy intermediate and enhances electron transfer from the metal surface to the carbonyl C thus facilitating the C–C coupling on certain metals like Cu.

### 3.9 Co-Reaction Between Aldehydes on Metal Surfaces

Strong heat of adsorption of conjugated aromatic aldehydes on the surface Cu has interesting implications during the simultaneous ECH of two different aldehydes and we briefly discuss these results in this section. First, let us focus on the coreaction of benzaldehyde and pentanal on Cu/C. Figure 8 presents the concentration profiles of reactants and products as a function of reaction time during the coreaction of benzaldehyde and pentanal on Cu/C. The concentration profiles during their individual reactions (i.e., without the other coreactant) under the same reaction conditions are also shown for comparison.

**Figure 8:**
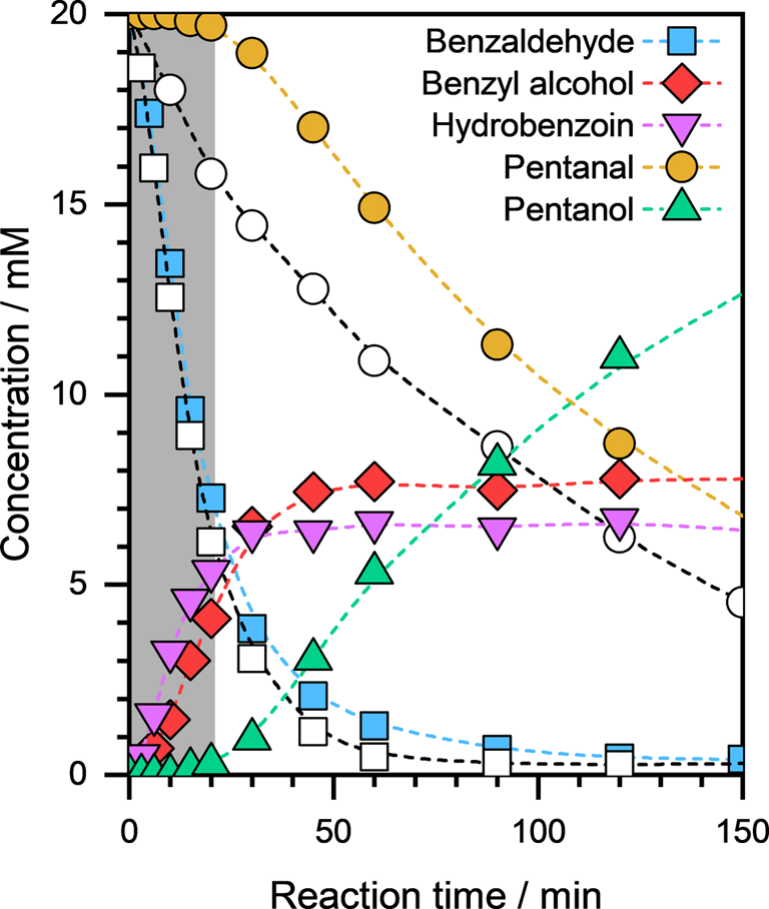
Concentration profiles of reactants and products, as a function of reaction time, during the coreaction (filled symbols) of benzaldehyde and pentanal on Cu/C. The concentration profiles during the individual reactions of benzaldehyde and pentanal are shown as open symbols. Reaction conditions: ∼10 mg catalyst, ∼20 mM organic substrate, *E*_ext_ = −0.5 V, ∼1.5 M sodium acetate/acetic acid buffer solution (pH ≈ 4.6), ambient temperature and pressure. The dashed lines are visual guides.

Notably, in the coreaction experiment, we observed the C–C coupling product of benzaldehyde but not of pentanal in addition to the formation of the respective C=O hydrogenation products. We noticed an unusual phenomenon during the course of the reaction—although benzaldehyde conversion started immediately at the start of the reaction, pentanal conversion was suppressed at initial reaction times (denoted by the gray zone; Figure 8). Pentanal conversion started only after ∼20 min of reaction when a substantial fraction of benzaldehyde had been converted, and as a result, benzaldehyde concentration in the electrolyte solution became low. We also observed similar behavior during the coreaction of benzaldehyde with other aliphatic aldehydes, viz., butanal and heptanal (see Figure S9). In both cases, the conversion of the aliphatic aldehyde was suppressed initially and only started once the benzaldehyde concentration was low.

This unusual behavior during the coreaction of benzaldehyde with pentanal suggests that the surface of Cu is initially saturated with benzaldehyde (or its hydrogenated derivatives), and the surface coverage of the pentanal is negligible. As the reaction proceeds, the surface coverage of benzaldehyde decreases as its concentration decreases, which allows pentanal to adsorb on the surface and be subsequently hydrogenated to pentanol. The relative surface coverages of benzaldehyde and pentanal agree well with the experimentally measured Δ*H*_ads_ of benzaldehyde (−145 kJ·mol^–1^) and pentanal (−40 kJ·mol^–1^) on Cu/C (see Figure 7). This difference in the surface coverages of benzaldehyde and pentanal on Cu/C was further evidenced by the different FEs observed during their ECH reactions. A high Faradaic efficiency during benzaldehyde ECH on Cu/C (∼94%; see Figure 1a) indicates high organic coverage, while a relatively low FE in the case of pentanal ECH on Cu/C (∼54%; see Figure 1b) suggests low coverage under the investigated reaction conditions.

To further investigate this phenomenon, we performed another coreaction experiment in which we reintroduced benzaldehyde to the electrolyte solution after ∼90 min reaction time. The concentration profiles of benzaldehyde and pentanal during this experiment, as a function of reaction time, are presented in Figure 9. We can see that the pentanal conversion was suppressed initially (indicated by the gray zone; Figure 9) and only began after benzaldehyde concentration was substantially reduced. However, and remarkably, upon the reintroduction of ∼20 mM equivalent benzaldehyde to the electrolyte solution at ∼90 min reaction time, benzaldehyde started converting immediately. The pentanal conversion was suppressed again, while benzaldehyde was being converted (i.e., between 90 and 110 min, indicated by the yellow zone; Figure 9). Pentanal started converting only when the benzaldehyde concentration was low again, i.e., after ∼110 min. Based on these results, we infer that upon its reintroduction, benzaldehyde replaced pentanal from the surface of the catalyst (due to its high −Δ*H*_ads_), thus suppressing pentanal conversion.

**Figure 9:**
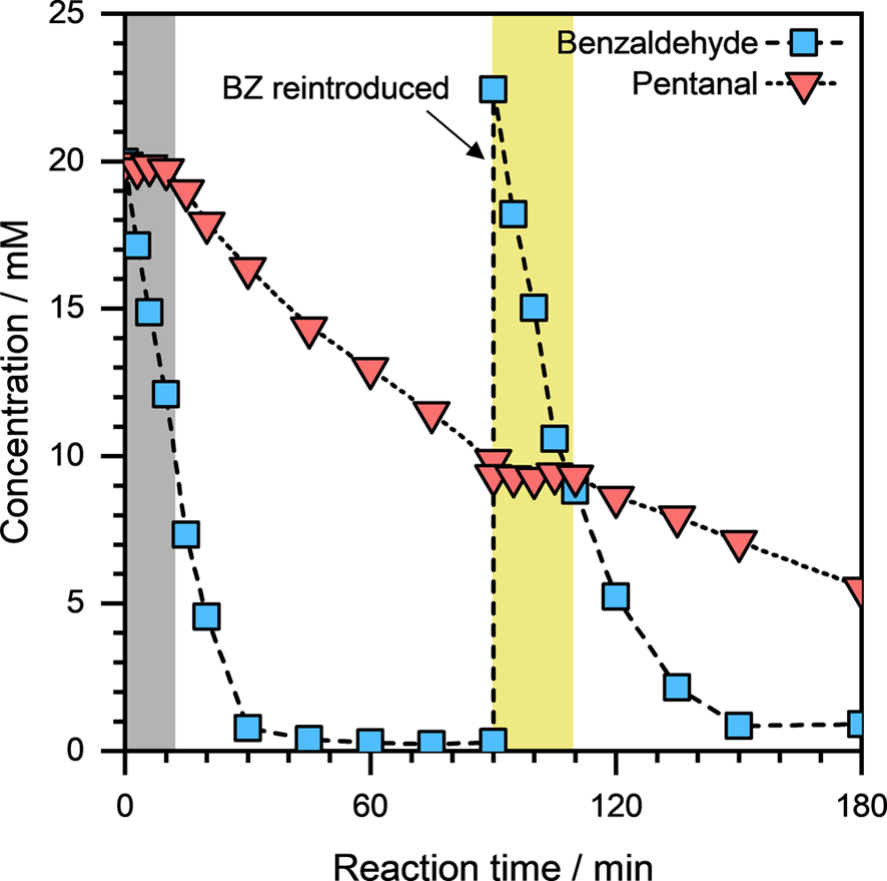
Concentration profiles of benzaldehyde and pentanal, as a function of reaction time, during the ECH of benzaldehyde and pentanal on Cu/C. benzaldehyde (20 mM equivalent) was reintroduced into the electrolyte at ∼90 min reaction time. Reaction conditions: ∼10 mg catalyst, ∼20 mM organic substrate, *E*_ext_ = −0.5 V, ∼1.5 M sodium acetate/acetic acid buffer solution (pH ≈ 4.6), ambient temperature and pressure. The dashed and dotted lines are visual guides.

Combining the coreaction experiment results, we can now describe the coreaction of benzaldehyde and pentanal (or other aliphatic aldehydes) on Cu/C in terms of two distinct phases: (i) an initial phase (corresponding to the first 20 min under these reaction conditions) when only benzaldehyde conversion takes place, and (ii) a second phase (that starts after ∼20 min) when primarily pentanal conversion takes place. The surface of the catalyst is saturated with benzaldehyde in the first phase due to its significantly higher −Δ*H*_ads_ compared to that of pentanal. In the second phase, the benzaldehyde concentration becomes low; this lower concentration decreases the surface coverage of benzaldehyde, thus allowing pentanal to adsorb on the surface and to be subsequently converted to pentanol.

We also performed the coreaction of benzaldehyde and pentanal on catalysts that did not promote the C–C coupling reaction, i.e., Pd/C and Ru/C. The corresponding concentration profiles of reactants and products, as a function of reaction time, are presented in Figure S10. Interestingly, during the coreactions of benzaldehyde and pentanal on Pd/C and Ru/C, we did not observe any suppression of pentanal conversion at the beginning of the reaction. The conversion of both coreactants started immediately at the beginning of the reaction. This concurrent conversion indicates that both aldehyde species are coadsorbed on the metal surface, which agrees well with the low −Δ*H*_ads_ of benzaldehyde on Pd/C and Ru/C. Finally, we investigated the coreaction of pentanal on Cu/C with other cyclic aldehydes that did not undergo C–C coupling, i.e., cyclohexenal and cyclohexanal. The corresponding concentration profiles of reactants and products are presented in Figure S11. During the coreaction of these cyclic aldehydes with pentanal, we again observed that the conversion of both coreactants started immediately. The concurrent conversion indicates that both aldehydes are adsorbed simultaneously on Cu. It is also noteworthy that the adsorption enthalpies of cyclohexanal and pentanal on Cu/C were similar (∼37 kJ·mol^–1^ for cyclohexanal and ∼43 kJ·mol^–1^ for pentanal; see Figure 7). Due to similar −Δ*H*_ads_ values of cyclohexanal and pentanal, we expect cyclohexanal and pentanal to compete for the available sites, resulting in their coadsorption and, therefore, concurrent conversion on Cu/C.

## 4 Conclusion

ECH of several aliphatic and aromatic aldehydes on different carbon-supported base metals (Cu, Ni and Co) and noble metals (Pd, Pt, Ru, Au and Rh) highlights the interesting property of Cu to promote electroreductive C–C coupling during the ECH of conjugated aromatic aldehydes (like benzaldehyde and furfural). Au/C and Co/C also catalyze C–C coupling to some degree but with lower rates and lower Faradaic efficiencies. Other metals primarily catalyze hydrogenation of the C=O functional group to form the corresponding alcohols. C–C coupling products were also not observed on Cu/C during the ECH of (i) aliphatic aldehydes like pentanal, (ii) cyclic nonaromatic aldehydes like cyclohexanal, (iii) nonconjugated aromatic aldehydes like hydrocinnamaldehyde, and (iv) conjugated nonaromatic aldehydes like crotonaldehyde.

For C–C coupling to occur on a metal surface, the following mechanistic conditions must be met. (i) First H addition to the carbonyl oxygen must be the preferred pathway; preferential first H addition to the carbonyl carbon will saturate it and inhibit subsequent C–C coupling. (ii) The second H addition step must be the kinetically rate-determining step; a fast second H addition will consume the hydroxy intermediate and form primarily the alcohol product. (iii) The intrinsic activation barrier for C–C coupling step must be lower than that for the second H addition step; a high barrier for the C–C bond formation will also inhibit the C–C coupling pathway. The C–C coupling observed during the ECH of conjugated aromatic aldehydes on Cu is facilitated by a strong adsorption of the planar organic substrate on the surface of the metal, and the conjugation between the aromatic ring and the C=O group, which stabilizes the surface hydroxy intermediate and facilitates charge transfer from the surface.

First-principles molecular simulations show that C–C coupling is suppressed during the ECH of nonaromaticaldehydes like cyclohexanal and pentanal on Cu(111) due to fast second H addition step. In the case of benzaldehyde ECH on Ru(0001), the preferential first H addition to the carbonyl C (facilitated by the lower electrophilicity of the carbonyl oxygen) prevents the subsequent C–C bond formation. Lastly, the C–C coupling exhibits significantly higher transition state barrier on Ru(0001) compared to Cu(111) due to relatively suppressed overall charge transfer from the metal surface to the adsorbed intermediate.

Strong adsorption of conjugated aromatic aldehydes on the surface Cu also has interesting implications during the simultaneous ECH reaction of two different aldehydes. When coreacting benzaldehyde with other aliphatic aldehydes, only benzaldehyde conversion takes place in the initial phase. Conversion of the aliphatic aldehyde only begins once benzaldehyde concentration becomes low, which decreases its surface coverage, thus allowing for the competing aldehyde to adsorb on the surface and to be subsequently converted.

## Supplementary Material



## Notes

Additional experimental details, computational methods, supplementary tables, and supplementary figures (PDF)
