# Supplementary material for: On the Mechanism of Electrocatalytic Carbon–Carbon Coupling of Conjugated Aromatic Aldehydes on Cu Cathodes
Source: ACS Catal. 2025 May 26;15(11):9762–75. doi: 10.1021/acscatal.4c08004 (PMC12150260; doi:10.1021/acscatal.4c08004)
Supplement: Supplementary file 1 [file cs4c08004_si_001.pdf]

Supporting Information for

On the Mechanism of Electrocatalytic Carbon–Carbon Coupling of Conjugated Aromatic Aldehydes on Cu Cathodes

Hongwen Chen,[a] M. Ussama,[a,b] Jayendran Iyer[a,b], Sungmin Kim,[c] M. Ali Haider,[a,b,d] Rachit Khare,[a]* and Johannes A. Lercher[a,c]*

[a]Department of Chemistry and Catalysis Research Center, Technical University of Munich, 85748 Garching, Germany

[b]Renewable Energy and Chemicals Laboratory, Department of Chemical Engineering, Indian Institute of Technology Delhi, 110016 New Delhi, India

[c]Institute for Integrated Catalysis, Pacific Northwest National Laboratory, 99354 Richland, Washington, United States

[d]Indian Institute of Technology Delhi–Abu Dhabi, Khalifa City B, MZ29 Abu Dhabi, UAE

*Corresponding authors: rachit.khare@tum.de, johannes.lercher@ch.tum.de

Table of Contents

S1. Supplementary Tables 3

S2. Supplementary Figures 12

S3. Computational Methods 25

S4. Additional Calculation Details 27

References 29

S1. Supplementary Tables

Table S1. Metal precursors and conditions (temperature and duration) for N_2 and H_2 treatment for the synthesis of different carbon-supported metal catalysts.

Catalyst	Precursor	N_2 treatment	H_2 treatment
Cu/C	$Cu(CH_3COO)_2$	673 K, 4 h	623 K, 4 h
Ni/C	$Ni(NO_3)_2$	673 K, 4 h	673 K, 4 h
Co/C	$Co(NO_3)_2{\cdot}6H_2O$	523 K, 2 h	523 K, 2 h
Ru/C	$RuCl_3{\cdot}xH_2O$	673 K, 4 h	523 K, 3 h
Pd/C	$Pd(CH_3COO)_2$	453 K, 2 h	523 K, 2 h
Rh/C	$Rh(NO_3)_3{\cdot}xH_2O$	673 K, 3 h	473 K, 1 h
Au/C	$AuCl_3{\cdot}3H_2O$	673 K, 2 h	573 K, 2 h

Table S2. Faradaic efficiencies towards benzyl alcohol, hydrobenzoin, cyclohexanemethanol, and H_2 formation during the ECH on benzaldehyde on various carbon-supported metals. Reaction conditions: 10 mg catalyst, 20 mM benzaldehyde, $E_{ext} =$ –0.5 V *versus* RHE, 1.5 M sodium acetate/acetic acid buffer solution ($pH \approx$ 4.6), ambient temperature and pressure.

**Catalyst**	**Faradaic efficiency / %**			
	**Benzyl alcohol**	**Hydrobenzoin**	**Cyclohexanemethanol**	**H_2**
Cu/C	45	49	-	6
Ni/C	56	-	-	44
Co/C	82	-	-	18
Ru/C	18	-	-	82
Pd/C	58	-	-	42
Pt/C	11	-	-	89
Rh/C	24	-	1.4	74
Au/C	26	-	-	74

Table S3. Faradaic efficiencies towards pentanol and H_2 formation during the ECH on pentanal on various carbon-supported metals. Reaction conditions: 10 mg catalyst, 20 mM pentanal, $E_{ext} =$ –0.5 V *versus* RHE, 1.5 M sodium acetate/acetic acid buffer solution ($pH \approx$ 4.6), ambient temperature and pressure.

Catalyst	Faradaic efficiency / %	
	Pentanol	H_2
Cu/C	54	46
Ni/C	28	72
Ru/C	19	81
Pd/C	3.2	97
Pt/C	2.2	98
Rh/C	0.85	99

Table S4. Faradaic efficiencies towards C=O hydrogenation products, C–C coupling products, and H_2 during the ECH of different aliphatic and cyclic aldehydes on Cu/C. Reaction conditions: 10 mg catalyst, 20 mM organic substrate, $E_{ext} =$ –0.5 V versus RHE, 1.5 M sodium acetate/acetic acid buffer solution ($pH \approx$ 4.6), ambient temperature and pressure.

**Organic Substrate**	**Faradaic efficiency / %**		
	**C=O hydrogenation**	**C–C coupling**	**H_2**
Propanal	9.7	-	90
Butanal	33	-	67
Pentanal	58	-	42
Heptanal	90	-	10
Hydrocinnamaldehyde	98	-	2.5
Cyclohexenal	88	-	12
Cyclohexanal	87	-	13

Table S5. Faradaic efficiencies towards the formation of C=O hydrogenation products, C–C coupling products, “*other*” products, and H_2 during the ECH of various conjugated aromatic aldehydes on Cu/C. Reaction conditions: 10 mg catalyst, 20 mM organic substrate, $E_{ext} = -0.5$ V *versus* RHE, 1.5 M sodium acetate/acetic acid buffer solution ($pH \approx$ 4.6), ambient temperature and pressure.

**Organic Substrate**	**Faradaic efficiency / %**			
	**C=O hydrogenation**	**C–C coupling**	**Others**	**H_2**
Benzaldehyde	49	44	-	7
4-Methylbenzaledhyed	23	30	1.3	46
4-Methoxybenzaldehyde	19	29	17	34
Furfural	49	3.1	34	14
3-Thiophenecarbonxaldehyde	7.2	25	-	68
Acetophenone	1.8	4.8	1.9	92

Table S6. Faradaic efficiencies towards benzyl alcohol and hydrobenzoin formation during the ECH on benzaldehyde on various carbon-supported metals in alkaline media. Reaction conditions: 10 mg catalyst, 20 mM benzaldehyde, $E_{ext} =$ –0.7 V versus RHE for Co and Au; –0.5 V versus RHE for other metals, 1 M NaOH/NaCl electrolyte solution ($pH \approx$ 11.3), ambient temperature and pressure.

**Catalyst**	**Faradaic efficiency / %**		
	**Benzyl alcohol**	**Hydrobenzoin**	**H_2**
Cu/C	15	84	0.78
Ni/C	-	-	100
Co/C	13	35	52
Ru/C	51	-	49
Pd/C	97	-	2.9
Pt/C	91	-	9.1
Rh/C	72	-	28
Au/C	8.1	44	48

Table S7. Faradaic efficiencies towards the formation of C=O hydrogenation products, C–C coupling products, "*other*" products, and H_2 during the ECH of various conjugated aromatic aldehydes on Cu/C in alkaline conditions. Reaction conditions: ~10 mg catalyst, ~20 mM organic substrate, $E_{ext} =$ –0.5 V *versus* RHE, ~1 M NaOH/NaCl electrolyte solution ($pH \approx$ 11.3), ambient temperature and pressure.

**Organic Substrate**	**Faradaic efficiency / %**			
	**C=O hydrogenation**	**C–C coupling**	**Others**	**H_2**
Benzaldehyde	17	82	0	1.2
4-Methylbenzaledhyde	49	47	0	3.6
4-Methoxybenzaldehyde	40	53	6.1	1.0
Furfural	51	13	31	5.1
3-Thiophenecarbonxaldehyde	1.5	73	4.8	20
Acetophenone	15	25	58	1.7
Pentanal	89	-	-	11
Cyclohexanal	92	-	-	8.0

Table S8. Bader charges on the carbonyl carbon and oxygen atoms of benzaldehyde and crotonaldehyde molecules adsorbed on the Cu(111) and Ru(0001) surface.

Surface	Substrate	Bader charge	
		Carbonyl C	Carbonyl O
Cu(111)	Benzaldehyde	0.68	–1.51
Ru(0001)	Benzaldehyde	0.43	–1.32
Cu(111)	Crotonaldehyde	0.32	–1.25

Table S9. Net Bader charge on the hydroxy intermediate (ArCHOH*) of benzaldehyde ECH on Cu(111) and Ru(0001) surfaces.

Surface	Bader charge
Cu(111)	–0.67
Ru(0001)	–0.59

S2. Supplementary Figures

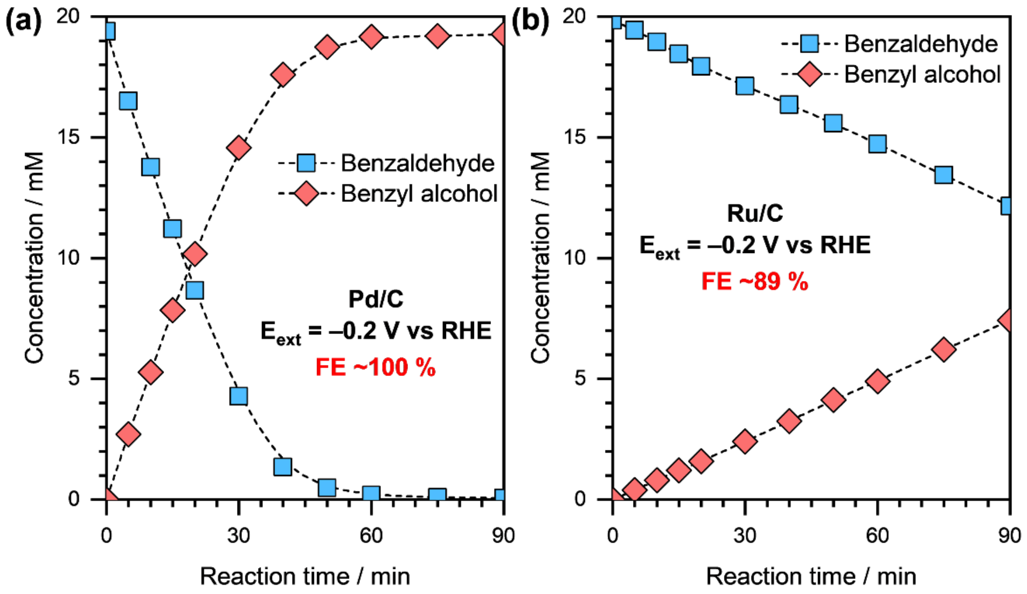

Figure S1. Concentration profiles of reactants and products, as a function of reaction time, during the ECH of benzaldehyde on (a) Pd/C and (b) Ru/C at E_{ext} = –0.2 V. Reaction conditions: 10 mg catalyst, 20 mM benzaldehyde, E_{ext} = –0.2 V *versus* RHE, 1.5 M sodium acetate/acetic acid buffer solution ($pH \approx$ 4.6), ambient temperature and pressure. The dashed lines are visual guides.

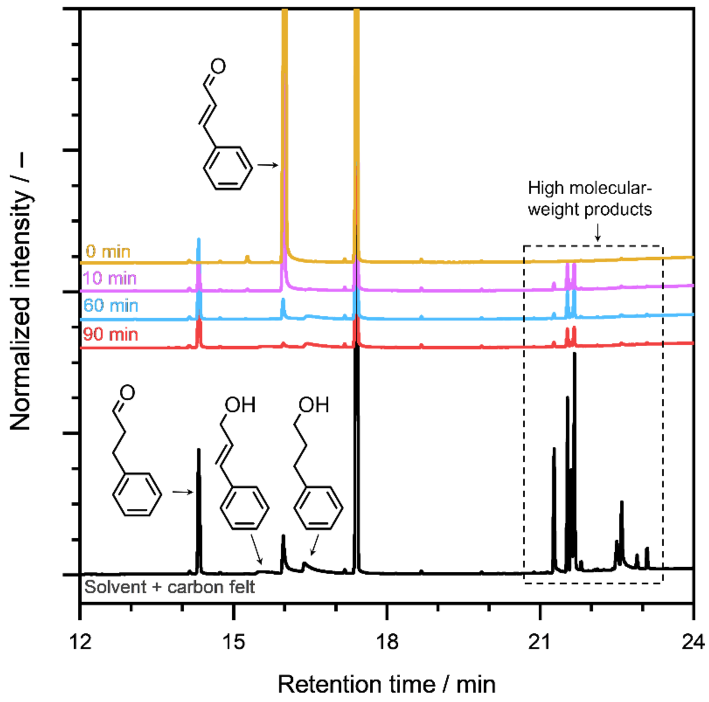

Figure S2. GC chromatograms of organics extracted from (i) the electrolyte solution at different reaction times during cinnamaldehyde ECH on Cu/C, and (ii) the electrolyte solution, carbon felt, and the spent catalyst at the end of the reaction. Reaction conditions: 10 mg catalyst, 20 mM cinnamaldehyde, $E_{ext} =$ –0.5 V *versus* RHE, 1.5 M sodium acetate/acetic acid buffer solution ($pH \approx$ 4.6), ambient temperature and pressure.

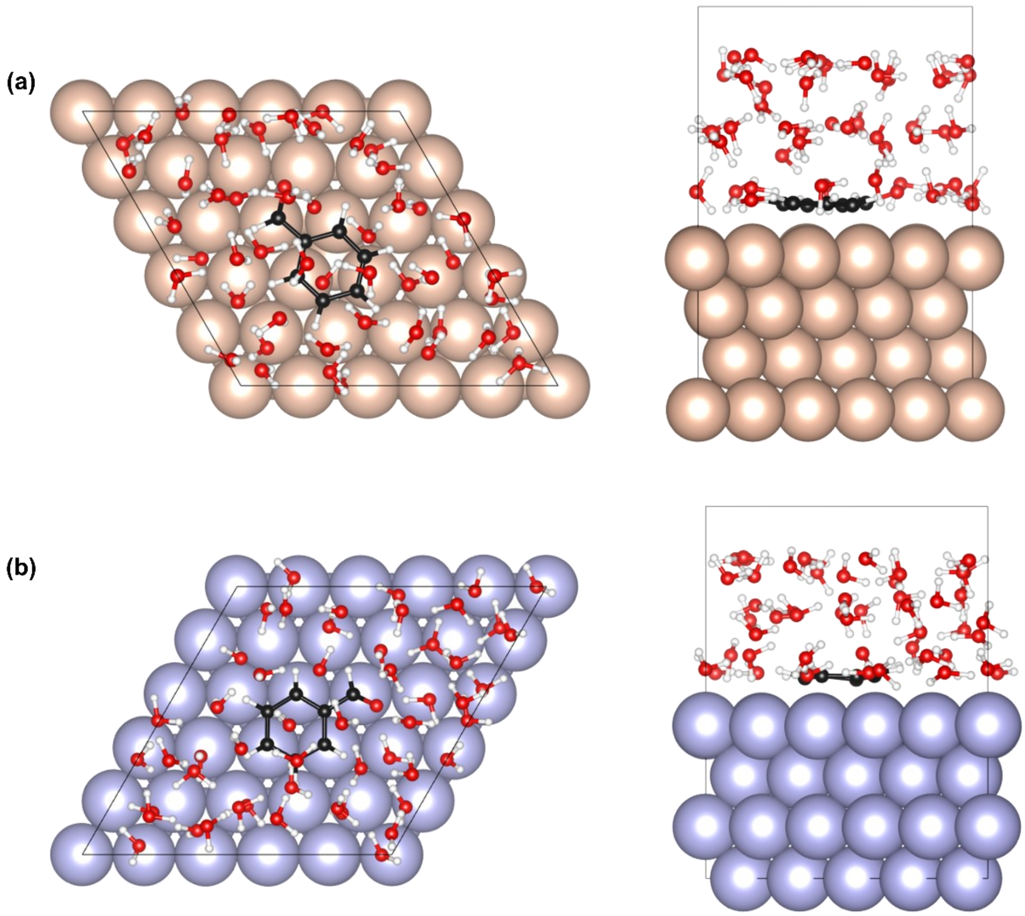

Figure S3. Top view (left panels) and side view (right panels) of the DFT-optimized geometries of the adsorbed benzaldehyde molecule on (a) Cu(111) and (b) Ru(0001) surfaces. Cu, orange; Ru, purple; C, black; O, red; H, white. The electrode-electrolyte systems comprised 100 metal atoms, one benzaldehyde molecule, 48 explicit water molecules, a proton (H^+), and a corresponding electron to simulate electrochemical hydrogenation conditions.

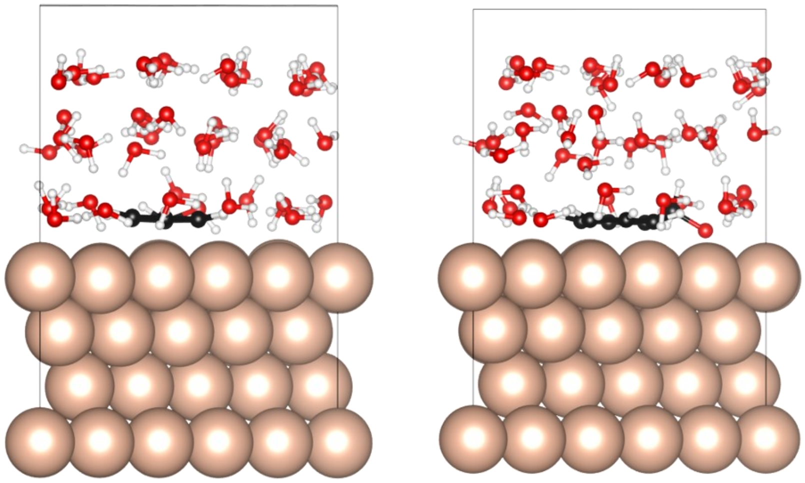

Figure S4. DFT-optimized geometries (side views) of the adsorbed hydroxy intermediate (left panels) and adsorbed alkoxy intermediate (right panels) of benzaldehyde ECH on the Cu(111) surface. Cu, orange; C, black; O, red; H, white. The electrode-electrolyte systems comprised 100 Cu atoms, the organic substrate, 48 explicit water molecules, a proton (H^+), and a corresponding electron to simulate the electrochemical hydrogenation conditions.

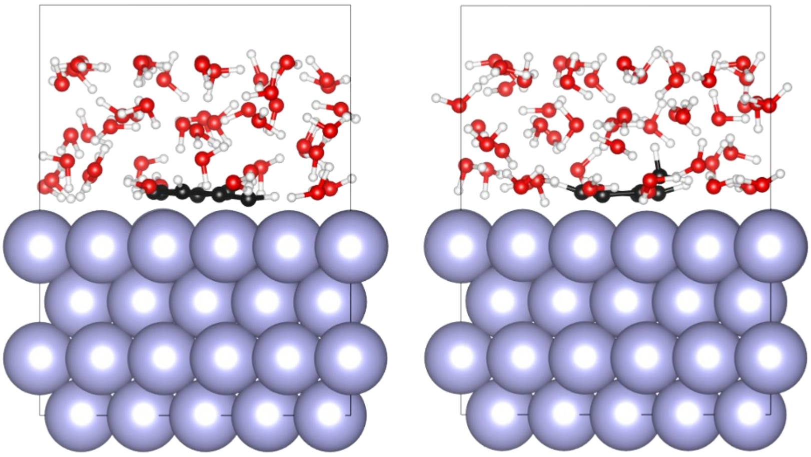

Figure S5. DFT-optimized geometries (side views) of the adsorbed hydroxy intermediate (left panel) and adsorbed alkoxy intermediate (right panel) of benzaldehyde ECH on the Ru(0001) surface. Ru, purple; C, black; O, red; H, white. The electrode-electrolyte systems comprised 100 Ru atoms, the organic substrate, 48 explicit water molecules, a proton (H^+), and a corresponding electron to simulate the electrochemical hydrogenation conditions.

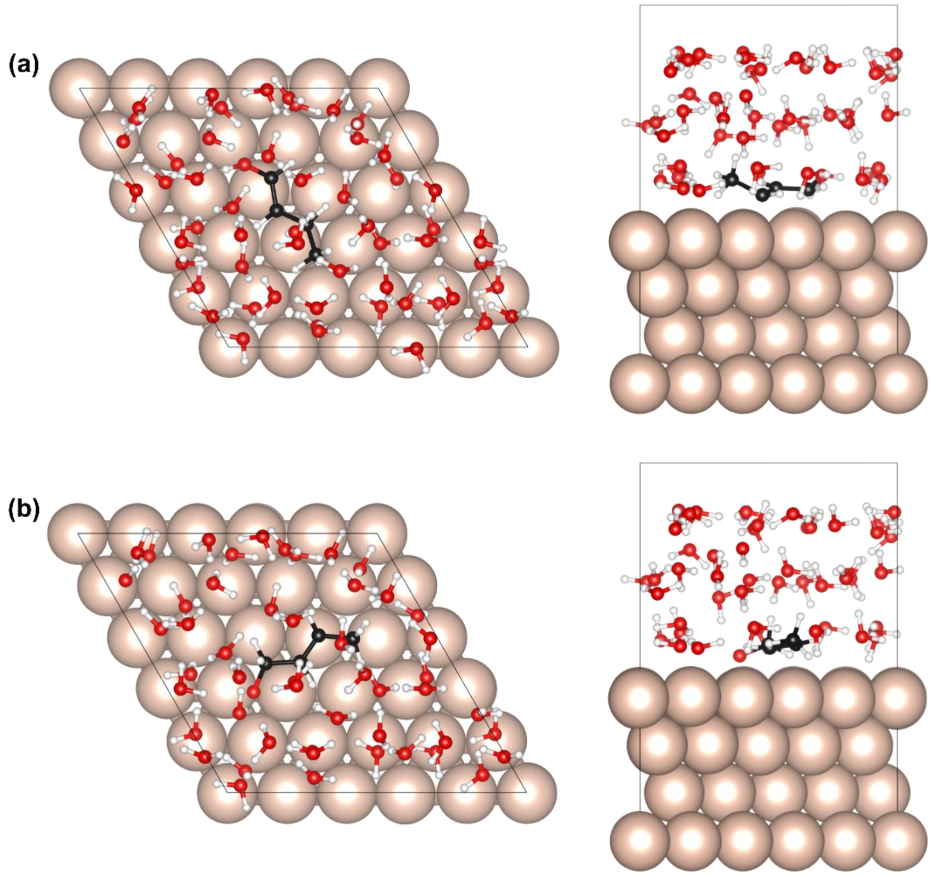

Figure S6. Top views (left panels) and side views (right panels) of the DFT-optimized geometries of the (a) adsorbed hydroxy intermediate and (b) adsorbed alkoxy intermediate of crotonaldehyde ECH on the Cu(111) surface. Cu, orange; C, black; O, red; H, white. The electrode-electrolyte systems comprise 100 metal atoms, an organic substrate, 48 explicit water molecules, a proton (H^+), and a corresponding electron, to simulate electrochemical hydrogenation conditions.

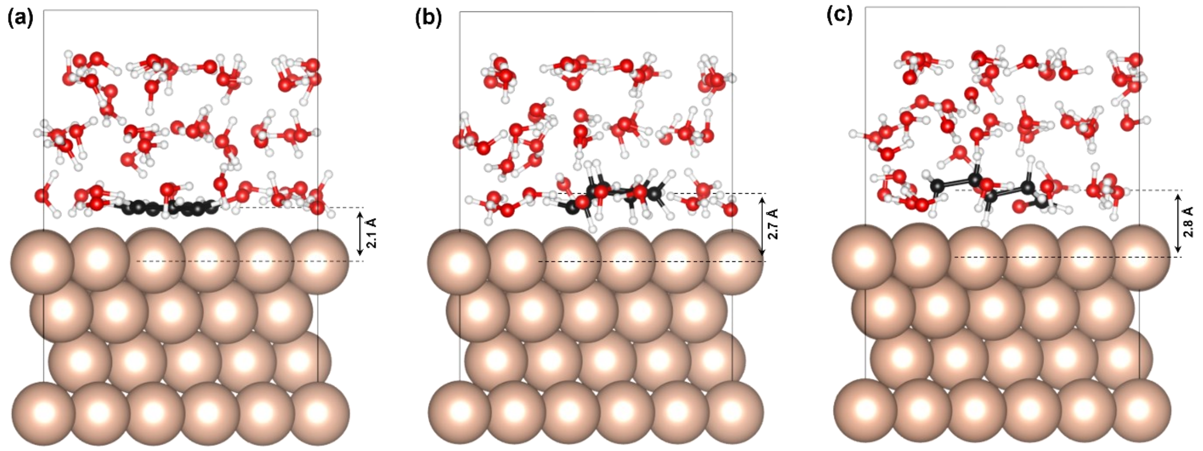

Figure S7. DFT-optimized geometries (side view) of the adsorbed (a) benzaldehyde, (b) cyclohexanal, and (c) pentanal on the Cu(111) surface. Cu, orange; C, black; O, red; H, white. The electrode-electrolyte systems comprise 100 metal atoms, the organic substrate, 48 explicit water molecules, a proton (H^+), and a corresponding electron to simulate the electrochemical hydrogenation conditions.

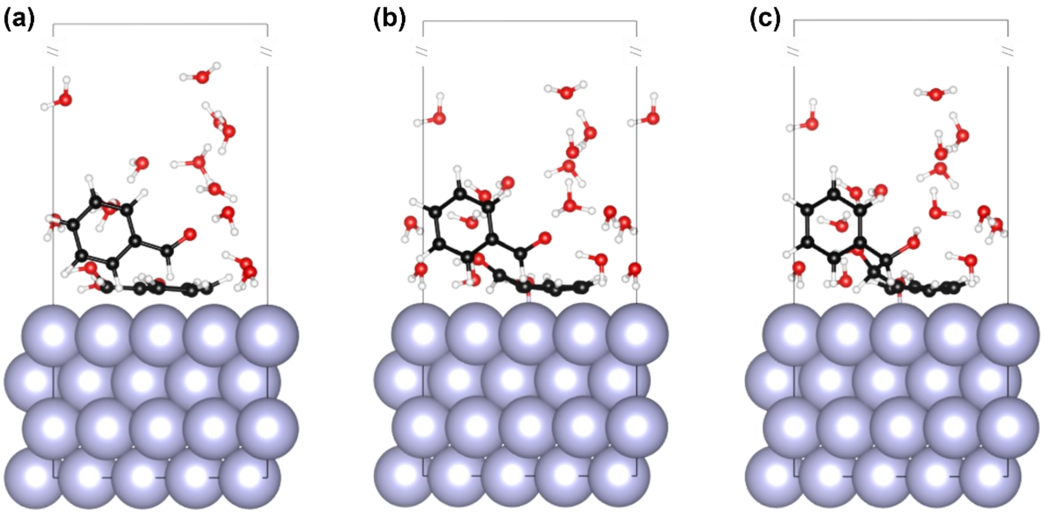

Figure S8. DFT-optimized geometries (side views) of the (a) initial state, (b) transition state, and (c) final state of the C–C bond formation step between a surface hydroxy intermediate and a physisorbed benzaldehyde molecule to form HB*. The simulations were performed on a Ru(1000) surface. Ru, purple; C, black; O, red; H, white. The electrode–electrolyte system comprised 64 Ru atoms, organic substrate, 16 explicit water molecules, a proton (H+), and a corresponding electron, to simulate electrochemical hydrogenation conditions.

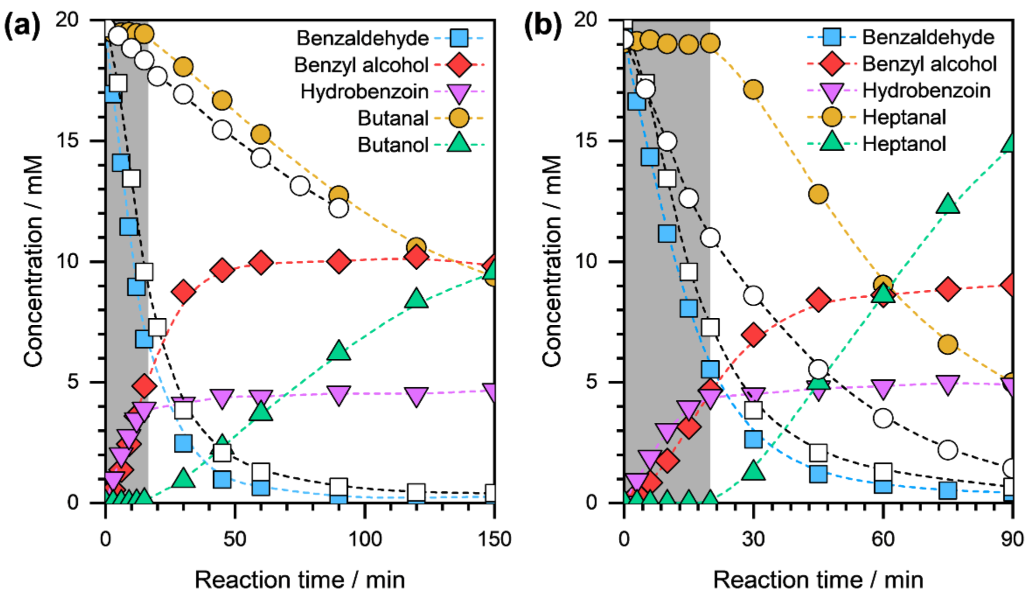

Figure S9. Concentration profile of reactants and products as a function of reaction time during the co-reaction (closed symbols) of (a) benzaldehyde with butanal, and (b) benzaldehyde with heptanal. The concentration profiles during the individual reactions are shown as open symbols. Reaction conditions: 10 mg catalyst, 20 mM organic substrate, $E_{ext} = -0.5$ V vs RHE, 1.5 M sodium acetate/acetic acid buffer solution ($pH \approx 4.6$), ambient temperature and pressure. The dashed lines are guides to the eye.

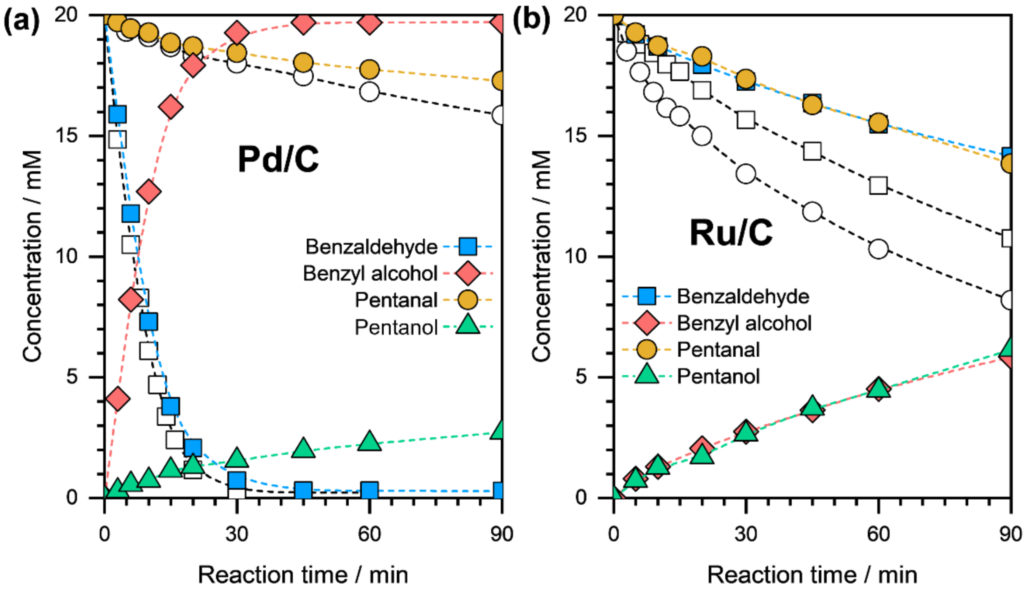

Figure S10. Concentration profiles of reactants and products, as a function of reaction time, during the ECH of benzaldehyde and pentanal on (a) Pd/C and (b) Ru/C. The concentration profiles during individual reactions of benzaldehyde and pentanal are also shown (as open symbols) for comparison. Reaction conditions: 10 mg catalyst, 20 mM organic substrate, E_{ext} = –0.5 V vs RHE, 1.5 M sodium acetate/acetic acid buffer solution ($pH \approx$ 4.6), ambient temperature and pressure. The dashed lines are visual guides.

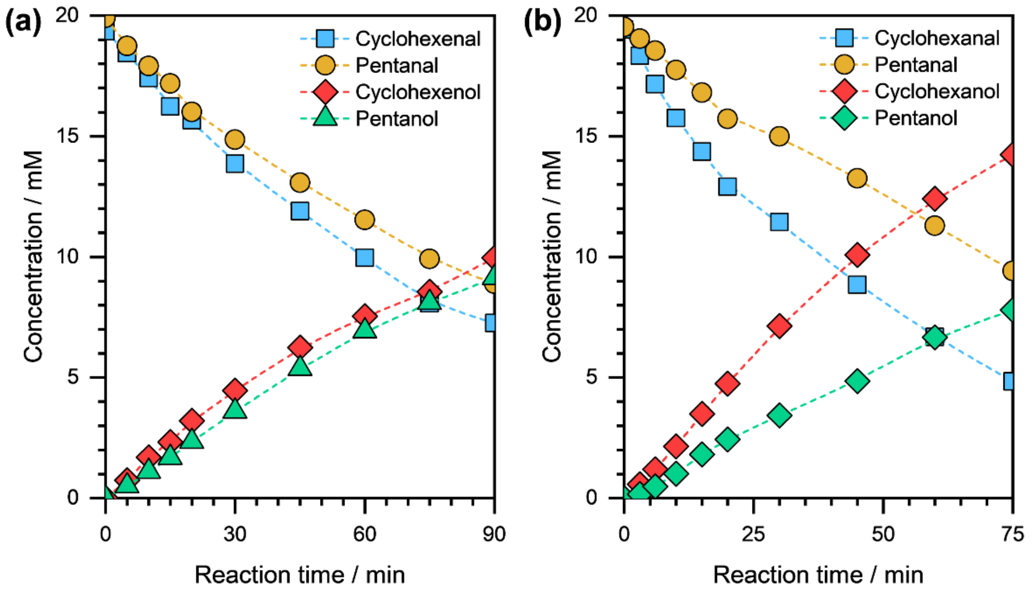

Figure S11. Concentration profiles of reactants and products, as a function of reaction time, during the co-reaction of (a) cyclohexenal and pentanal and (b) cyclohexanal and pentanal on Cu/C. Reaction conditions: ~10 mg catalyst, ~20 mM organic substrate, E_{ext} = –0.5 V vs RHE, 1.5 M sodium acetate/acetic acid buffer solution ($pH \approx$ 4.6), ambient temperature and pressure. The dashed lines are visual guides.

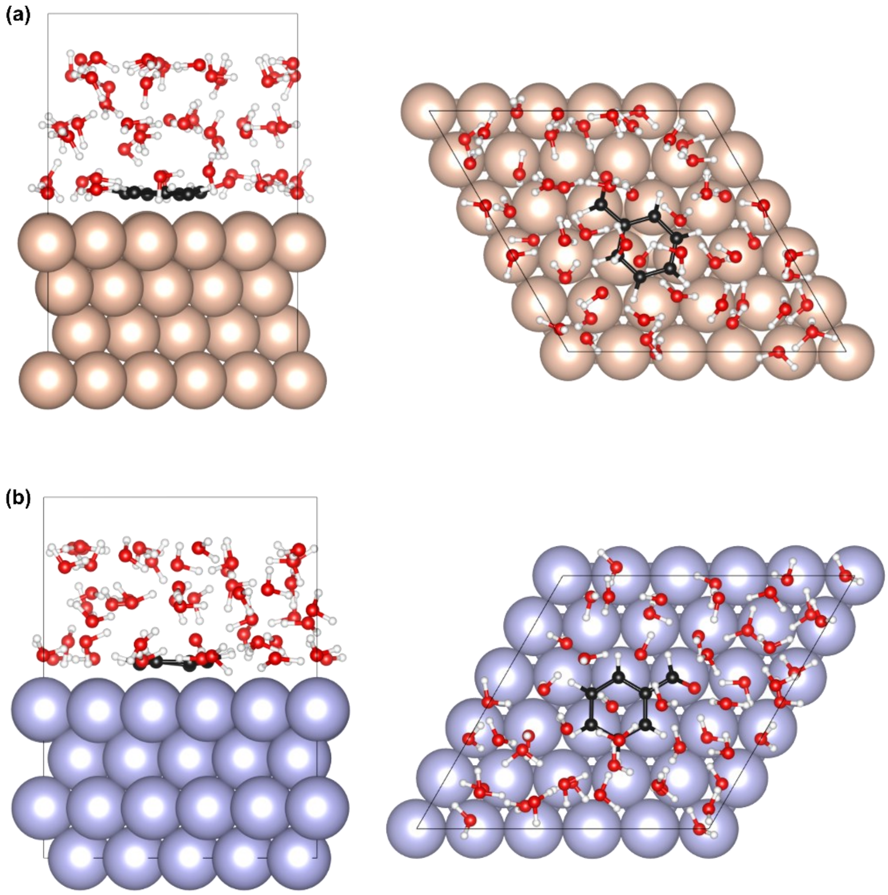

Figure S12. Side view (left panels) and top view (right panels) showing benzaldehyde adsorbed on (a) Cu(111) surface and (b) Ru(0001) surface with 48 explicit water molecules and a proton (H^+). Cu, orange; Ru, purple; C, black; O, red; H, white.

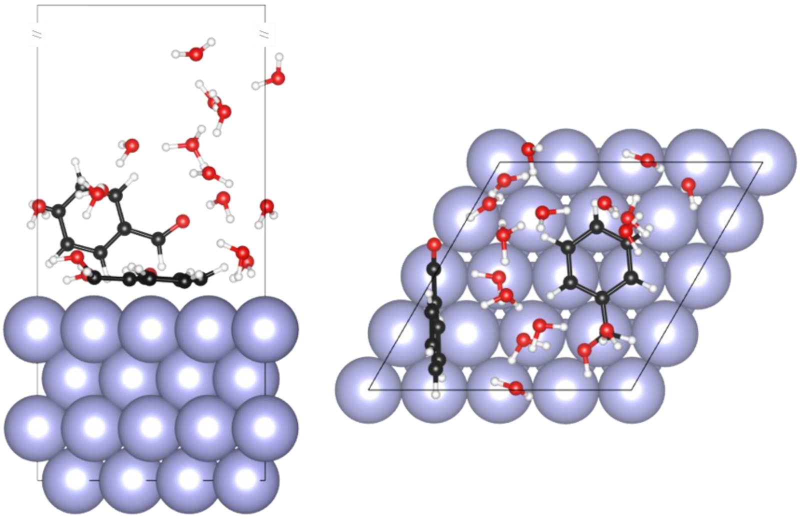

Figure S13. Side view (left panel) and top view (right panel) showing benzaldehyde adsorbed on the Ru(0001) surface with 16 explicit water molecules and a proton (H^+). Ru, purple; C, black; O, red; H, white.

S3. Computational Methods

First-principles molecular simulations were performed using the CP2K electronic structure and molecular dynamics software package (v2022.2).[1] The simulations were performed on a Cu(111) or a Ru(0001) surface, each composed of four atomic layers. During the electronic structure calculations, the bottom two layers were constrained, while the top two layers were allowed to relax. The simulation box size was 12.78 × 12.78 × 16.26 $Å^3$ for Cu and 13.50 × 13.50 × 15.42 $Å^3$ for Ru. The volume of solvation layer above the metal surface was ~1420 $Å^3$ in both cases. To ensure a realistic solvation environment, 54 water molecules were initially placed in the vacuum region and equilibrated using *ab initio* molecular dynamics (AIMD) at room temperature, allowing them to reorganize and form structured solvation layers. Following equilibration, the organic substrate was introduced by removing six water molecules near the surface, ensuring appropriate system composition while maintaining the integrity of the structured water layers. The electrochemical conditions were simulated by adding a proton (H^+) to the water layer and a corresponding electron to the system. The final model electrode-electrolyte system comprised 100 atoms of Cu or Ru, 48 explicit water molecules, a proton (H^+), an organic substrate, and an electron. The top and side views of representative electrode-electrolyte systems for Cu and Ru electrodes are presented in **Figure S12**.

For electronic structure optimizations, a hybrid Gaussian and plane waves (GPW) method implemented in the Quickstep module of the CP2K software package was employed.[1-3] The expanded electron density in the auxiliary plane wave basis set was moderated with an energy cutoff of 400 Ry. The 4d5s electrons of Ru, 3d4s electrons of Cu, 2s2p electrons of O and C, and the 1s electrons of H were considered as the valence states and were expanded using the optimized double-ζ plus polarization quality Gaussian basis sets (MOLOPT-SR DZVP),[4] while the ionic cores were represented by norm-conserving Goedecker-Teter-Hutter (GTH) pseudopotentials.[5-7] The Perdew-Burke-Ernzerhof (PBE)[8] density functional was applied in conjunction with DFT-D3 dispersion corrections described by Grimme *et al.* to account for exchange correlation effects within the generalized gradient approximation (GGA).[9] Matrix diagonalization was used to solve the Kohn−Sham equations, and the electronic structure convergence criteria was set to 1.0 × 10^{-6} a.u. for the electronic self-consistent field (SCF)

loops. Additionally, SCF convergence was accelerated by applying Fermi-Dirac smearing at an electronic temperature of 300 K.

Nudged-elastic band (NEB) calculations to determine the transition-state barrier for C–C coupling reaction on Ru(0001) surface were performed on a periodic electrode-electrolyte interface model using the Vienna *ab initio* simulation package (VASP) with a plane wave basis set.[10-13] These calculations were performed on a 4 × 4× 4 slab of Ru(0001) surface. The top two layers of the slab were allowed to relax during the simulations, while the bottom two layers were fixed representing bulk Ru. The electrode-electrolyte interface was modelled with an implicit solvation model using 16 explicit H_2O molecules, one proton (H^+) and the reaction intermediates above the Ru(0001) surface. A vacuum of at least 10 Å above the water layer. The top and side views of a representative electrode-electrolyte system are presented in **Figure S13**.

In these calculations, the single-electron wavefunctions were truncated at an energy cut-off of 400 eV. The k-points were sampled from a grid size 2 × 2 × 1 in the Brillouin zone. The partial occupancies were set for each orbital using the Methfessel-Paxton scheme to smoothen the wavefunctions.[14] The core electrons were treated with the frozen-core approximations using projector augmented-wave (PAW) method. The electron self-interaction in the Hartree potential were treated using revised Perdew-Burke-Ernzerhof (PBE) exchange correlation functional with the dispersion corrections using Becke-Johnson (BJ) damping scheme.[15, 16] The open-source *VASPsol* package was used to implement implicit solvent in all *ab initio* simulations with spin-polarized calculations.[17] The water and intermediate configurations on Ru(0001) were sampled from AIMD simulations performed using a canonical ensemble at a constant temperature of 300 K. The transition state (TS) for the C–C coupling step was identified using the climbing image-nudged elastic band technique (cI-NEB) with eight intermediate images between the initial state (IS) and the final state (FS) along the reaction coordinate with the energy and force convergence set at 1×10^{-5} eV and 0.1 eV·Å^{-1}, respectively.[18]

S4. Additional Calculation Details

The conversion of a reactant i at any given time t was estimated by

$$\chi_i(t) = \frac{n_i(t)}{n_i^0}$$

where $n_i(t)$ is the amount of reactant detected at time t and n_i^0 is the initial amount of reactant in the electrolyte.

Similarly, the yield of a product j with respect to a reactant i at any given time t was estimated by

$$Y_j(t) = \frac{n_j(t)}{n_i^0}$$

where $n_j(t)$ is the amount of reactant j detected at time t and n_i^0 is the initial amount of reactant i in the electrolyte solution.

The initial ECH rate of a product i (in terms of H consumed per gram metal) was calculated from the initial slope (m_i) of the product i formed *versus* time plot using the following equation

$$r_i = \frac{m_i \times \epsilon_i}{w_{cat} \times x_{metal}}$$

where w_{cat} is the amount of catalyst, x_{metal} is the metal loading on the catalyst (as weight fraction), and ϵ_i is the number of H atoms required to form the product i.

H_2 formation was indirectly estimated from the difference in total electron consumption (Q_{total}) and electron consumption towards the formation of organic products ($Q_{organics}$). The amount of H_2 formed (n_{H_2}) was then calculated using the following equation.

$$n_{H_2} = \frac{Q_{H_2}}{2 \cdot F} = \frac{Q_{total} - Q_{organics}}{2 \cdot F}$$

where Q_{H_2} is the electron consumption towards H_2 formation, and F is the Faraday constant (= 96,485 C·mol^{-1}).

The Faradaic efficiency towards the formation of a product i was estimated by

$$FE_i = \frac{Q_i}{Q_{total}}$$

where Q_i is the electrons consumed towards formation of product i, and Q_{total} is the total electrons consumed.

The overall Faradaic efficiency towards organic conversion was estimated using the following equation

$$FE = \frac{Q_{organics}}{Q_{total}}$$

where Q_i denotes the electrons consumed towards organic conversion.

The Faradaic selectivity towards a specific organic product i was estimated using the following equation

$$FS_i = \frac{Q_i}{Q_{organics}}$$

References

(1) Kühne, T. D.; Iannuzzi, M.; Del Ben, M.; Rybkin, V. V.; Seewald, P.; Stein, F.; Laino, T.; Khaliullin, R. Z.; Schütt, O.; Schiffmann, F.; et al. CP2K: An electronic structure and molecular dynamics software package - Quickstep: Efficient and accurate electronic structure calculations. *The Journal of Chemical Physics* **2020**, *152* (19).

(2) VandeVondele, J.; Krack, M.; Mohamed, F.; Parrinello, M.; Chassaing, T.; Hutter, J. Quickstep: Fast and accurate density functional calculations using a mixed Gaussian and plane waves approach. *Computer Physics Communications* **2005**, *167* (2), 103-128.

(3) Lippert, B. G.; Parrinello, J. H.; Michele. A hybrid Gaussian and plane wave density functional scheme. *Molecular Physics* **1997**, *92* (3), 477-488.

(4) VandeVondele, J.; Hutter, J. Gaussian basis sets for accurate calculations on molecular systems in gas and condensed phases. *J Chem Phys* **2007**, *127* (11), 114105.

(5) Goedecker, S.; Teter, M.; Hutter, J. Separable dual-space Gaussian pseudopotentials. *Physical Review B* **1996**, *54* (3), 1703-1710.

(6) Hartwigsen, C.; Goedecker, S.; Hutter, J. Relativistic separable dual-space Gaussian pseudopotentials from H to Rn. *Physical Review B* **1998**, *58* (7), 3641-3662.

(7) Krack, M. Pseudopotentials for H to Kr optimized for gradient-corrected exchange-correlation functionals. *Theoretical Chemistry Accounts* **2005**, *114* (1), 145-152.

(8) Perdew, J. P.; Burke, K.; Ernzerhof, M. Generalized Gradient Approximation Made Simple. *Physical Review Letters* **1996**, *77* (18), 3865-3868.

(9) Grimme, S.; Antony, J.; Ehrlich, S.; Krieg, H. A consistent and accurate ab initio parametrization of density functional dispersion correction (DFT-D) for the 94 elements H-Pu. *The Journal of Chemical Physics* **2010**, *132* (15).

(10) Kresse, G.; Furthmüller, J. Efficiency of ab-initio total energy calculations for metals and semiconductors using a plane-wave basis set. *Computational Materials Science* **1996**, *6* (1).

(11) Kresse, G.; Furthmüller, J. Efficient iterative schemes for ab initio total-energy calculations using a plane-wave basis set. *Physical Review B* **1996**, *54* (16).

(12) Kresse, G.; Hafner, J. Ab initio molecular-dynamics simulation of the liquid-metal–amorphous-semiconductor transition in germanium. *Physical Review B* **1994**, *49* (20), 14251-14269.

(13) Kresse, G.; Hafner, J. Ab initio molecular dynamics for liquid metals. *Physical Review B* **1993**, *47* (1).

(14) Methfessel, M.; Paxton, A. T. High-precision sampling for Brillouin-zone integration in metals. *Physical Review B* **1989**, *40* (6), 3616-3621.

(15) Hammer, B.; Hansen, L. B.; Nørskov, J. K. Improved adsorption energetics within density-functional theory using revised Perdew-Burke-Ernzerhof functionals. *Physical Review B* **1999**, *59* (11), 7413-7421.

(16) Grimme, S.; Ehrlich, S.; Goerigk, L. Effect of the damping function in dispersion corrected density functional theory. *Journal of Computational Chemistry* **2011**, *32* (7), 1456-1465.

(17) Mathew, K.; Sundararaman, R.; Letchworth-Weaver, K.; Arias, T. A.; Hennig, R. G. Implicit solvation model for density-functional study of nanocrystal surfaces and reaction pathways. *The Journal of Chemical Physics* **2014**, *140* (8).

(18) Henkelman, G.; Uberuaga, B. P.; Jónsson, H. A climbing image nudged elastic band method for finding saddle points and minimum energy paths. *The Journal of Chemical Physics* **2000**, *113* (22), 9901-9904.
